# The opportunistic pathogen *Pseudomonas aeruginosa* exploits bacterial biotin synthesis pathway to benefit its infectivity

**DOI:** 10.1371/journal.ppat.1011110

**Published:** 2023-01-23

**Authors:** Yu Shi, Qin Cao, Jingdu Sun, Xiaofang Hu, Zhi Su, Yongchang Xu, Huimin Zhang, Lefu Lan, Youjun Feng

**Affiliations:** 1 Department of Microbiology, and Department of General Intensive Care Unit of the Second Affiliated Hospital, Zhejiang University School of Medicine, Hangzhou, Zhejiang, China; 2 State Key Laboratory of Drug Research, Shanghai Institute of Materia Medica, Chinese Academy of Sciences, Shanghai, China; 3 School of Pharmaceutical Science and Technology, Hangzhou Institute for Advanced Study, University of Chinese Academy of Sciences, Hangzhou, Zhejiang, China; 4 College of Animal Sciences, Zhejiang University, Hangzhou, Zhejiang, China; 5 Fuzhou Medical College of Nanchang University, Fuzhou, Jiangxi, China; 6 College of Life Science and Technology, Guangxi University, Nanning, Guangxi, China; 7 Cancer Center at Illinois, University of Illinois at Urbana-Champaign, Urbana, Illinois, United States of America; University of Washington, UNITED STATES

## Abstract

*Pseudomonas aeruginosa* is an opportunistic pathogen that predominantly causes nosocomial and community-acquired lung infections. As a member of ESKAPE pathogens, carbapenem-resistant *P*. *aeruginosa* (CRPA) compromises the limited therapeutic options, raising an urgent demand for the development of lead compounds against previously-unrecognized drug targets. Biotin is an important cofactor, of which the *de novo* synthesis is an attractive antimicrobial target in certain recalcitrant infections. Here we report genetic and biochemical definition of *P*. *aeruginosa* BioH (PA0502) that functions as a gatekeeper enzyme allowing the product pimeloyl-ACP to exit from fatty acid synthesis cycle and to enter the late stage of biotin synthesis pathway. In relative to *Escherichia coli*, *P*. *aeruginosa* physiologically requires 3-fold higher level of cytosolic biotin, which can be attributed to the occurrence of multiple biotinylated enzymes. The BioH protein enables the *in vitro* reconstitution of biotin synthesis. The repertoire of biotin abundance is assigned to different mouse tissues and/or organ contents, and the plasma biotin level of mouse is around 6-fold higher than that of human. Removal of *bioH* renders *P*. *aeruginosa* biotin auxotrophic and impairs its intra-phagosome persistence. Based on a model of CD-1 mice mimicking the human environment, lung challenge combined with systemic infection suggested that BioH is necessary for the full virulence of *P*. *aeruginosa*. As expected, the biotin synthesis inhibitor MAC13772 is capable of dampening the viability of CRPA. Notably, MAC13772 interferes the production of pyocyanin, an important virulence factor of *P*. *aeruginosa*. Our data expands our understanding of *P*. *aeruginosa* biotin synthesis relevant to bacterial infectivity. In particular, this study represents the first example of an extracellular pathogen *P*. *aeruginosa* that exploits biotin cofactor as a fitness determinant, raising the possibility of biotin synthesis as an anti-CRPA target.

## Author summary

Carbapenem-resistant *P*. *aeruginosa* (CRPA) is a recalcitrant member of critically-prioritized ‘ESKAPE’ pathogens, threatening global public health. Bacterial biotin synthesis is recognized as a promising druggable pathway. This study physiologically explained why cytosolic biotin level in *P*. *aeruginosa* is relatively-higher than that of *E*. *coli*. In addition to its gatekeeper role in biotin synthesis, *P*. *aeruginosa* BioH was also found to behave as a fitness determinant. The repertoire of biotin abundance was assigned to an array of mouse tissues and organ contents. Using a model of CD-1 mice that mimics the human plasma environment, lung challenge combined with systemic infection revealed that *P*. *aeruginosa* BioH benefits bacterial infectivity. Notably, the biotin synthesis inhibitor MAC13772 can interfere the production of pyocyanin, an important virulence factor in *P*. *aeruginosa*. Taken together, our findings provided metabolic insights into *P*. *aeruginosa* infection biology, raising the possibility of biotin synthesis as an anti-CRPA target.

## Introduction

Emergence and spread of antimicrobial resistance (AMR) become a serious challenge of global health concern. Before 2014, 700,000 peoples per year died of global AMR infections, and annual 10 million of deaths is estimated to be associated with drug-resistant infections unless AMR is tackled [1]. The Gram-negative bacterium, *Pseudomonas aeruginosa*, is a ubiquitous opportunistic pathogen that frequently causes nosocomial and community-acquired infections. In fact, *P*. *aeruginosa* is a predominant causative of chronic lung infections in cystic fibrosis (CF) patients, with infection in up to 70% of adult CF patients [2–4]. It is also noted that rapid dissemination of carbapenem-resistant *P*. *a**eruginosa* (CRPA) is a major threat in clinic settings, compromising the limited therapeutic options [5, 6]. Therefore, *P*. *aeruginosa* is included by the World Health Organization (WHO) into an ‘ESKAPE’ panel of pathogens, which are critically prioritized to tackle AMR [1]. The successful infection of *P*. *aeruginosa* largely relies on the production of virulence-associated components (e.g., pyocyanin [7] and biofilms [8, 9]) and its regulatory networks including two-component systems [10, 11] and quorum sensing [9, 12]. Essentially, *P*. *aeruginosa* dictates certain metabolic fluxes to circumvent host immune defenses and antimicrobial killing, and thus benefits its colonization and proliferation during infections [13, 14]. In light that the complex interplay of bacterial metabolisms occurs at infection sites, deep understanding of *P*. *aeruginosa* metabolic states [exemplified with biotin [15–18] and lipoic acid [19, 20]] might offer alternative strategies to prevent and intervene the severe infections caused by this extracellular pathogen [14, 21].

The water-soluble B vitamin cofactor, biotin is widely-distributed in all the three domains of life [22, 23]. As a sulfur-containing, seven-carbon fatty acid derivative, this covalently-linked coenzyme plays critical roles in the CO_2_ transfer between carboxylation and decarboxylation reactions involved in central metabolisms, including fatty acid biosynthesis, glucogenesis, and amino acid catabolism [24, 25]. To date, six biotin-requiring enzymes are identified [26], namely i) acetyl-CoA carboxylase (ACC) [25, 27]; ii) propionyl-CoA carboxylase (PCC) [28]; iii) pyruvate carboxylase (PC) [29, 30]; iv) 3-methylcrotonyl-CoA (MCC) [31]; v) geranyl-CoA carboxylase (GCC) [32]; and vi) urea carboxylase (UC) [33]. Among them, ACC is regarded as a promising drug target against numbers of diseases (such as obesity plus type 2 diabetes) [26, 34]. Mutations in either PCC or MCC can cause metabolic disorders in human neurodevelopment [35–37]. It is also noted that biotin, which serves as an essential micro-nutritional element, is generally produced by most of bacteria, fungi, and plants, whereas not in mammals and birds [38]. Increasingly-accumulated evidence supported that biotin is a nutritional virulence factor for certain bacterial pathogens [16–18, 39] and that its metabolism behaves as an attractive target against severe bacterial infections [40–43]. Intriguingly, biotin is found to mediate host-microbiome interaction, and regulates intestinal stem mitosis [44]. This is in part (if not all) consistent with the clinical observation that the maintenance of gut microbiota biotin metabolism benefits the metabolic status of patients with severe obesity [45]. Therefore, it seems very true that biotin is an old vitamin but possesses new roles in the crosstalk of nutritional sensing with human health and/or metabolic diseases.

The pathway of *de novo* biotin synthesis is composed of two stages: primary stage and late stage [23]. The early stage is engaged in the generation of pimeloyl-ACP (and/or CoA) thioester, a C7-fatty acyl precursor of biotin [46, 47], while the late step is dedicated to assembling the fused rings of biotin [38]. Unlike the late route that is catalyzed successively by four conserved enzymes BioF/A/D/B [38], the primary step differs dramatically in diverse microorganisms [48, 49]. So far, no less than three distinct mechanisms are programed to produce the cognate biotin precursor (i.e., pimeloyl-ACP (or CoA) species). Among them, the prototypical machinery refers to ‘BioC-BioH’ path that produces the pimeloyl-ACP (C7-ACP) species [47, 50]. It has also been reported that the ‘BioI-BioW’ bipartite model gives pimeloyl-CoA thioester [51, 52], and BioZ ligates malonyl-ACP with glutaryl-CoA, yielding pimeloyl-ACP, a *bona fide* precursor for biotin synthesis [48, 49]. Different from BioC that is a SAM-dependent methyltransferase [46], BioH appears to be a rather promiscuous demethylase [47, 50]. Furthermore, an arsenal of BioH isoenzymes are detected in diverse microbes, including *Haemophilus* BioG [53], *Francisella* BioJ [17, 54], and *Helicobacter* BioV [55]. Evolutionary and structural studies also elucidate that the BioH isoenzymes are grouped into distinct subclades within the family of α/β-hydrolases [50, 53, 54], which further underscores the diversity of bacterial biotin synthesis pathway.

**Fig 1:**
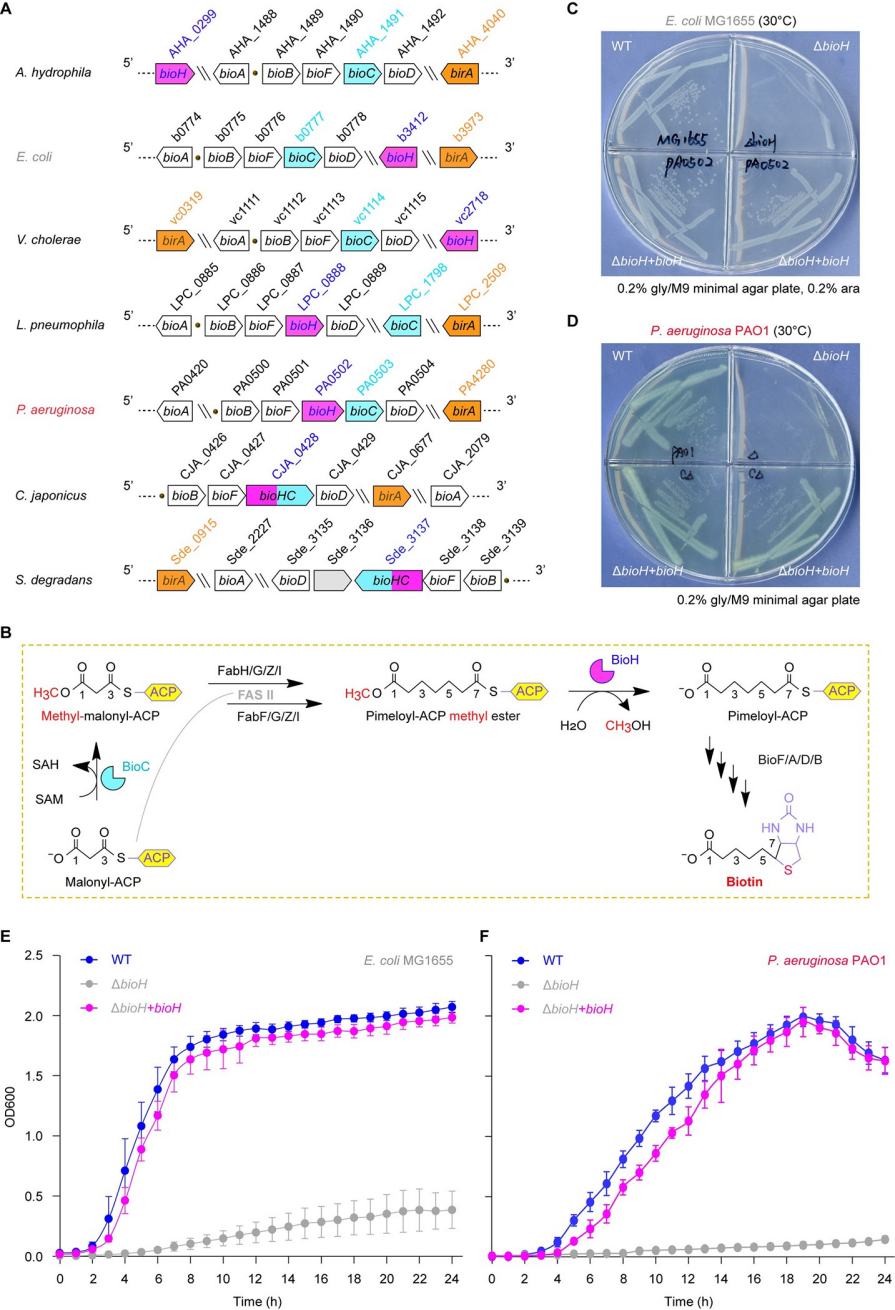
A role of PA0502 in biotin synthesis pathway **A.** Genetic context of *Pseudomonas aeruginosa bioH* (PA0502) and its counterparts in certain microorganisms **B.** A model for ‘BioC-BioH’ path of biotin synthesis in *P*. *aeruginosa*. **C.** Expression of PA0502 allows growth of the biotin auxotroph of *E*. *coli* Δ*bioH* on the non-permissive condition of M9 minimal medium. **D.** The removal of PA0502 renders *P*. *aeruginosa* biotin auxotrophic. A representative result from three independent experiments (in panels **C** & **D**) is given. **E.** Growth curves of the *E*. *coli* Δ*bioH* strains with or without plasmid-borne *bioH* (PA0502). **F.** Growth analyses of *P*. *aeruginosa* Δ*bioH* mutant and its complemented strain. As for plotting growth curves, the data is expressed in means ± standard deviation (SD) of three independent experiments (in panels **E** & **F**). Designations: SAM, S-adenosylmethionine; SAH, S-Adenosylhomocysteine; ACP, acyl carrier protein; FASII, Fatty acid synthesis type II; FabH, β-ketoacyl-ACP synthase III; FabF, β-ketoacyl-ACP synthase II; FabG, 3-keto-acyl-ACP reductase; FabZ, β-hydroxy-acyl-ACP dehydratase; FabI, enoyl-ACP reductase; BioC, O-methyltransferase of malonyl-ACP; BioH, demethylase of pimeloyl-ACP methyl ester; BioF, 8-amino-7-oxononanoate synthase; BioA, 7,8-diaminopelargonic acid synthase; BioD, dethiobiotin synthetase; BioB, biotin synthase.

In this study, we aimed to examine the contribution of biotin synthesis to *P*. *aeruginosa* virulence. Genomic context and functional assignment suggested the existence of an “BioC-BioH” early pathway for biotin synthesis in *P*. *aeruginosa* (**Fig 1A** and **1B**). Unlike the paradigm BioH that is encoded by the free-standing *bioH* in *E*. *coli*, the counterpart of *P*. *aeruginosa* is clustered with *bioC* in an operon (**Fig 1A**) [56]. Cao and coworkers reported the *in vitro* enzymatic activity of *P*. *aeruginosa* BioH and pointed out its expression is independent of genomic context [56]. However, the question whether genetic removal of the gatekeeper render *P*. *aeruginosa* biotin auxotrophic awaits answering. More importantly, the role of biotin synthesis in *P*. *aeruginosa* infectivity is poorly understood. Here we report that this is the case, and close the knowledge gap in the context of biotin metabolism along with bacterial virulence. This constitutes an additional example for biotin as a limited/nutritional virulence factor.

## Results and discussion

### Diversified distribution of ‘*bioC*-*bioH*’ pair

The machinery of ‘BioC-BioH’ is a canonical route engaged in the formation of pimeloyl-ACP methyl ester (M-C7-ACP), a cognate precursor for biotin synthesis (**Fig 1A** and **1B**). A modified type II fatty acid synthesis (FASII) pathway is hijacked by BioC and BioH [47]. In this case, BioC O-methyltransferase introduces a SAM-derived methyl moiety to the ω-carboxyl group of malonyl-CoA, giving an atypical primer malonyl-CoA methyl ester dedicated to two rounds of FASII cycles [46]. As a result, the methyl group of M-C7-ACP is no longer required, and eliminated by BioH demethylase to produce C7-ACP, a primer for late step of biotin synthesis catalyzed successively by BioF/A/D/B (**Fig 1B**) [24, 47]. Genome analysis unveils that the genetic organization of ‘*bioC*-*bioH*’ loci varies dramatically in diverse *bioH*-carrying bacterial species (**Fig 1A**). Totally, four types of genomic contexts are assigned to ‘*bioC*-*bioH*’ pair. The paradigm pattern refers to the *bioA*/*BFCD* operon of *E*. *coli* and *Vibrio* species, along with the free-standing *bioH* and *birA* (**Fig 1A**).

It is noted that BirA acts as a bifunctional biotin operon repressor having an additional activity of biotin-[acetyl-CoA-carboxylase subunit B, AccB] ligase [57, 58]. In the Legionnaires diseases-causing bacterium, *Legionella pneumophila*, it gives a *bioA*/*BFHD* cluster, almost identical to the prototypic version of *bioA*/*BFCD*. The only exception lies in that *bioC* of *E*. *coli* is replaced with BioH of *L*. *pneumophila*, and *vice versa* (**Fig 1A**). It represents a second mode for ‘*bioC*-*bioH*’ distribution. In contrast to the paradigmatic version, the *bio* cluster of *P*. *aeruginosa* seems to be more domesticated because that *bioH* (PA0502) is also integrated, forming a unique *bioBFHCD* operon (**Fig 1A**). The genetic organization of *bioBFHCD* cluster can assure synergistic expression of BioC and BioH, the two enzymes responsible for primary step of biotin synthesis, benefitting efficient production of biotin [56]. In this case, unlike the *bioA* of *E*. *coli* that is encoded in an operon, the counterpart of *Pseudomonas* remains scattered on the chromosome and is exempt from the regulation of BirA bifunctional repressor (**Fig 1A**). This can be the third style of ‘BioC-BioH’ organization. Intriguingly, a similar scenario is seen in the soil bacterium *Cellvibrio japonicus*, and the carbohydrates-degrading marine bacterium *Saccharophagus degradans* (**Fig 1A**). However, *bioH* and *bioC* that are supposed to be two adjacent genes, appear to be fused into a single gene *bioHC* (**Figs 1A** and **S1**). Namely, they include CJA_0428 for *C*. *japonicus*, and Sde_3137 for *S*. *degradans* (**Fig 1A**). Here, the presence of fusion BioHC is provisionally termed as the fourth form of ‘BioC-BioH’ combination. Overall, the unexpected diversity is displayed among the genomic arrangement of bacterial ‘BioC-BioH’ paths.

### Essentiality of *bioH* (PA0502) for *P*. *aeruginosa* viability

**Fig 2:**
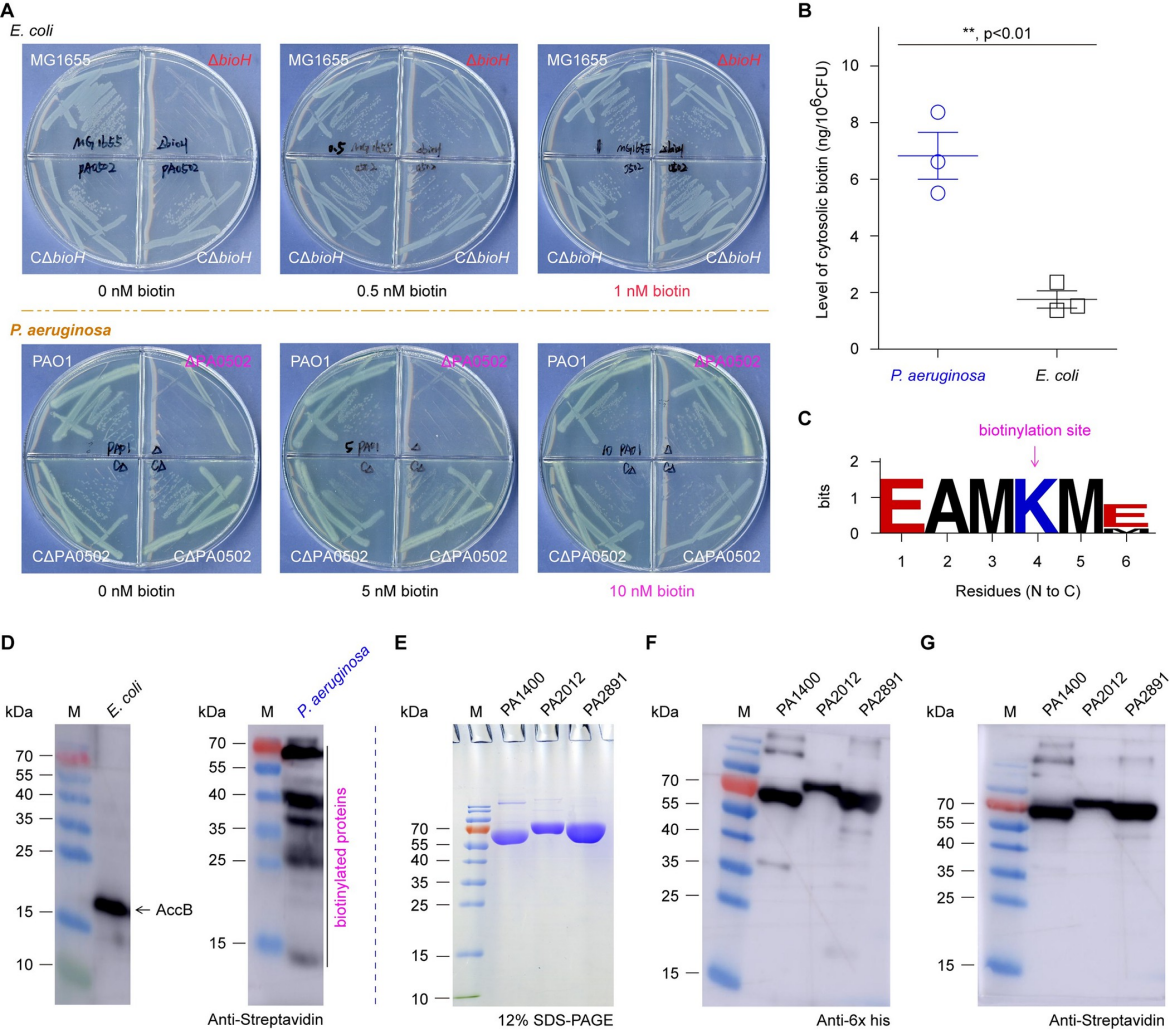
The requirement of biotin and protein biotinylation for *P*. *aeruginosa*. **A.** *P*. *aeruginosa* physiologically demands biotin at the level of 10 nM, 10-fold higher than that of *E*. *coli*. 0.5–1.0 nM biotin can restore growth of the biotin auxotrophic strain, *E*. *coli* Δ*bioH* mutant. In contrast, over 10 nM biotin is required to favor the appearance of *P*. *aeruginosa* Δ*bioH* mutant on the non-permissive growth condition lacking biotin. **B.** Biotin determination suggested that *P*. *aeruginosa* has much higher level of cytosolic biotin than *E*. *coli* does. Bacterial cultures in mid-log phase were sampled for the preparation of crude extracts used in cytosolic biotin determination. Data was expressed in an average ± standard deviations (SD). Each dot represented an independent measurement of biotin level. **C.** Sequence logo of biotinylation sites ubiquitously conserved in biotin-requiring enzymes of *P*. *aeruginosa*. Genome-wide mining of *P*. *aeruginosa* reveals five putative biotinylated enzymes, namely PA1400, PA2012, PA2891, PA4847, and PA5435 (**S1 Table**). **D.** Streptavidin blot analyses for crude extracts of *E*. *coli* and *P*. *aeruginosa*. Unlike *E*. *coli* displaying a well-known AccB subunit with biotinylation, the cytosol extract of *P*. *aeruginosa* is featuring with the presence of multiple biotinylated proteins. **E.** SDS-PAGE (12%) profile for three biotinylated enzymes (PA1400, PA2012, and PA2891). Three of five predictive biotinylated enzymes were overexpressed, purified, and judged with SDS-PAGE (12%). **F.** Western blot analysis of the three recombinant proteins (PA1400, PA2012, and PA2891) using anti-6x His primary antibody. **G.** Use of streptavidin blot to determine the biotin modification of the three recombinant proteins (PA1400, PA2012, and PA2891). Designations: CΔ*bioH*, a genetically-complemented strain of the *E*. *coli* Δ*bioH* mutant with *P*. *aeruginosa* PA0502; CΔPA0502, the genetically-complemented strain of the *P*. *aeruginosa* ΔPA0502 mutant carrying a plasmid pAK1900-borne PA0502.

Unlike BioC that determines methyl malonyl-CoA to enter FASII as a disguised primer, BioH functions as a gatekeeper enzyme of biotin synthesis in that it informs the resultant product M-C7-ACP to exit the ongoing FASII cycle via demethylation (**Fig 1B**). To test the role of *bioH* (PA0502) *in vivo*, we performed two sets of genetic systems (*E*. *coli* and *P*. *aeruginosa*). As expected, arabinose-induced expression of *bioH* enables the biotin auxotroph of *E*. *coli* Δ*bioH* mutant to grow in a chemically-defined M9 medium lacking biotin (**Fig 1C**). This is generally consistent with an observation by Cao and coworkers [56]. The deletion of *bioH* renders *P*. *aeruginosa* biotin auxotrophic, and plasmid pAK1900-borne PA0502 expression restores the impaired growth phenotype the Δ*bioH* mutant, at a comparable level of the parental strain PAO1 (**Fig 1D**). Similar scenarios were seen with the measurement of growth curves. Namely, i) the introduction of *bioH* into *E*. *coli* Δ*bioH* mutant confers the re-gain of robust growth in biotin-lacking liquid media (**Fig 1E**); and ii) genetic removal of *bioH* impairs bacterial viability of *P*. *aeruginosa* Δ*bioH* on non-permissive growth condition (**Fig 1F**). In fact, growth defection of Δ*bioH* mutants is restored by the presence of exogenous biotin (**Fig 2A**). Unlike *E*. *coli* that requires biotin at minimal level of 0.5–1.0 nM, the opportunistic pathogen *P*. *aeruginosa*, physiologically demands the addition of exogenous biotin at 10 to 20-fold higher level (**Fig 2A**). Combined with the earlier observations of Cao *et al*. [56], our genetic evidence underscores the physiological role of *bioH* in *P*. *aeruginosa* biotin synthesis.

### Physiological demand of biotin by *Pseudomonas*

An ELISA kit-based measurement revealed that cytosolic biotin in *P*. *aeruginosa* of mid-log phase is at over 3-fold higher level compared with that of *E*. *coli* (**Fig 2B**). It is thus reasonable to ask the question whether or not the much higher demand of biotin by *P*. *aeruginosa* is implicated into the existence of more biotin-requiring enzymes (**Fig 2A** and **2B**). To test this hypothesis, we conducted genome-wide search for biotinylated proteins. As a result, five hits of *P*. *aeruginosa* were returned, all of which feature with a conserved motif (EAM**K**ME) containing the constant lysine (K) residue biotinylated (**Fig 2C**). Namely, these enzymes include PA1400 (pyruvate carboxylase), PA2012 (α-subunit of methyl crotonyl-CoA carboxylase), PA2891 (α-subunit of geranyl-CoA carboxylase), PA4847 (AccB, biotin carboxyl carrier protein), and PA5435 (the probable transcarboxylase subunit) (**S1 Table**). To verify the bioinformatic output, we carried out streptavidin-based blot using bacterial crude extract. As expected, only one band of protein biotinylation was detected from the positive control strain, *E*. *coli* that is well-known in the biotinylated subunit of AccB (**Fig 2D**). It was noted that multiple bands of biotinylated proteins appear in our streptavidin blot for the crude extract derived from *P*. *aeruginosa* PAO1 (**Fig 2D**). Among them, three protein candidates (PA1400, PA2012, and PA2891) were selected for further consolidation of biotin modification. All the three recombinant enzymes were overexpressed and purified to homogeneity (**Fig 2E**). Their identities were respectively validated using western blot with anti-6x His primary antibody (**Fig 2F**). Meanwhile, streptavidin blot confirmed that they consistently contain biotinylated form (**Fig 2G**). The data benefits our understanding of physiological explanation for the relatively-high demand of biotin by *P*. *aeruginosa* (**Fig 2**). In fact, similar scenarios had already been seen with the plant pathogen, *Agrobacterium tumefaciens* [59] and the human pathogen surrogate, *Mycobacterium smegmatis* [60, 61]. Therefore, the accumulated evidence enabled us to raise the possibility that an arsenal of certain pathogens requires high-level of biotin for its central metabolism in an adaptation to the harsh host environments.

### Enzymatic activity of *P*. *aeruginosa* BioH

An N-terminal hexa-histidine (6x His)-tagged version of *P*. *aeruginosa* BioH (PA0502) protein was overexpressed and purified to homogeneity (**S2A Fig**). Using a Superdex 75 column, gel filtration analysis elucidated that the wild-type BioH protein is eluted at the position of ~12.5 ml (**S2B** and **S3A Figs**), which migrates relatively-slower than the fatty acid-binding protein FakB2 of a known monomer (~11.5 ml, ~33 kDa). This suggested that BioH of *P*. *aeruginosa* appears as a monomer (~25 kDa), which is largely consistent with the monomeric solution states of the paradigm *E*. *coli* BioH [50] and its isoenzyme BioJ [17, 54]. As described with BioJ [17], we also tested activity of *P*. *aeruginosa* BioH with its physiological substrate M-C7-ACP. The reaction mixture was separated with conformationally-sensitive gel of 0.5 M urea/17.5% PAGE (pH9.5), in which the product C7-ACP is supposed to migrate slower than its reactant M-C7-ACP due to the loss of methyl moiety [47]. Clearly, the *in vitro* activity of *P*. *aeruginosa* BioH behaves in a dose-dependent manner (**S2C Fig**). Of note, full activity of enzymatic demethylation can be reached when the *P*. *aeruginosa* BioH is added as low as 50 nM (**S2D Fig**). Subsequently, MALDI-TOF mass spectrometry was used to differentiate *P*. *aeruginosa* BioH reaction mixture. As predicted, the reactant M-C7-ACP exhibited a unique spectrum of 9002.281 m/z, close to its theoretical value of 9003.3 (**S2E Fig**), and the product of C7-ACP whose theoretical value is 8989.3 m/z was assigned with a distinct mass profile of 8988.772 m/z (**S2F Fig**). Collectively, along with the enzymatic analyses of Cao *et al*. [56], this study biochemically defined *Pseudomonas* BioH is a functional demethylase of M-C7-ACP.

### Catalytic triad of *P*. *aeruginosa* BioH

**Fig 3:**
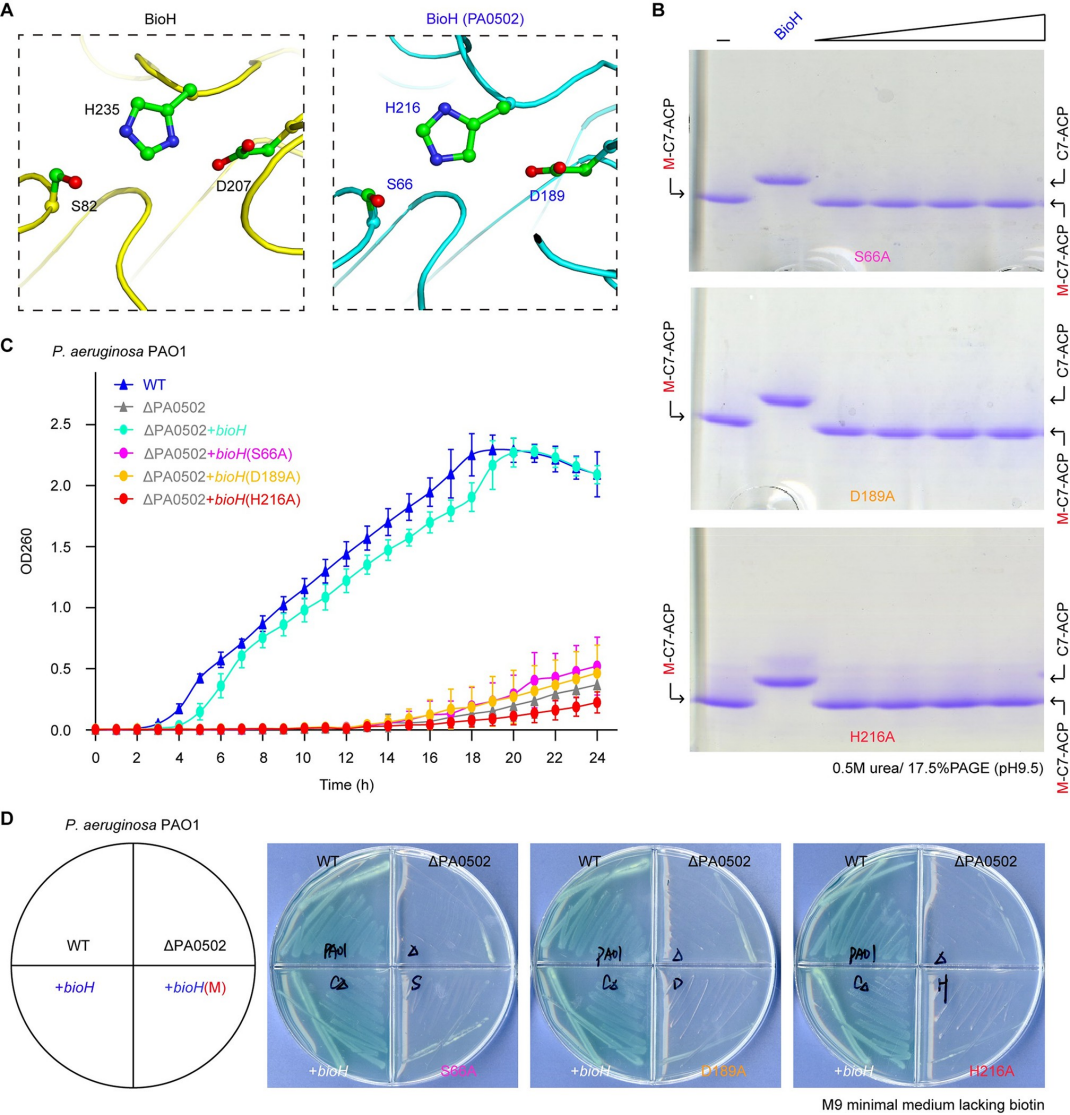
Functional characterization of BioH (PA0502) catalytic triad (S66, D189, and H216). **A.** Structural snapshot of BioH catalytic triad. The structure of *E*. *coli* BioH (PDB: 1M33) is generated with PyMol, and the structural model of PA0502 was predicted with Alpha-fold [79]. The catalytic triad of *E*. *coli* BioH consists of S82, D207, and H235. Equivalently, they denote S66, D189, and H216 in PA0502. **B.** The *in vitro* enzymatic assays reveal that none of three single mutants defective in catalytic triad is active. Namely, the three single mutants of PA0502 include S66A, D189A, H216A. The conformationally-sensitive gel of 0.5 M urea/17.5% PAGE (pH9.5) was applied to distinguish the product C7-ACP from its reactant M-C7-ACP. The minus symbol denotes no addition of PA0502. The positive control refers to the addition of wild-type PA0502 (50 nM). The triangle on right hand denotes varied level of PA0502 mutant (ranging from 0.5, 5, 50, to 500 nM). **C.** Growth curve analysis of *P*. *aeruginosa* PAO1 suggested that catalytic triad is essential for PA0502 activity. The graph was plotted with data collected from three independent measurements, and presented in an average ± standard deviations (SD). **D.** The alanine substitution of catalytic triad inactivates the ability of PA0502 to restore bacterial growth of the biotin auxotrophic strain ΔPA0502 on the non-permissive, biotin-deficient condition of M9 minimal medium.

BioH is a member of the α/β-hydrolase family with characteristics of a catalytic triad. Multiple sequence alignment of *P*. *aeruginosa* BioH with its homologues revealed the presence of three conserved resides forming a putative catalytic triad, namely S66, D189, and H216 (**Figs 3A**, **S1** and **S3A**). The predicted structure of *P*. *aeruginosa* BioH with AlphaFold enabled us to illustrate a catalytic triad of which the configuration is almost identical to the counterpart of the paradigm *E*. *coli* BioH (S82, D207, and H235, in **Fig 3A**). To test the function of this catalytic triad, we generated three single BioH mutants (S66A, D189A, and H216A, in **S3A Fig**), along with its wild-type whose identity was validated with mass spectrometry (**S3B Fig**). Except for the BioH mutant (H216A) that behaves as its wild-type to form a monomer, the remaining two mutated versions (S66A and D189A) are constantly eluted at the position of polymer in the analysis of size exclusion chromatography (**S3A Fig**). As expected, all the three mutants are enzymatically inactive with its cognate substrate M-C7-ACP, regardless of their solution structures (**Fig 3B**). Next, we examined the roles of these mutants *in vivo* by visualizing the viability of engineered *E*. *coli* carrying plasmid-borne PA0502 and its derivatives. Unlike its parental version PA0502, none of its three mutants with certain defection in catalytic triad can allow bacterial occurrence of the biotin auxotroph *P*. *aeruginosa* Δ*bioH* on the non-permissive condition of both defined M9 liquid media (**Fig 3C**) and M9 minimal agar plates (**Fig 3D**). Thus, this represents a functional proof of the catalytic triad of *P*. *aeruginosa* BioH.

### *P*. *aeruginosa* BioH enables biotin synthesis *in vitro*

**Fig 4:**
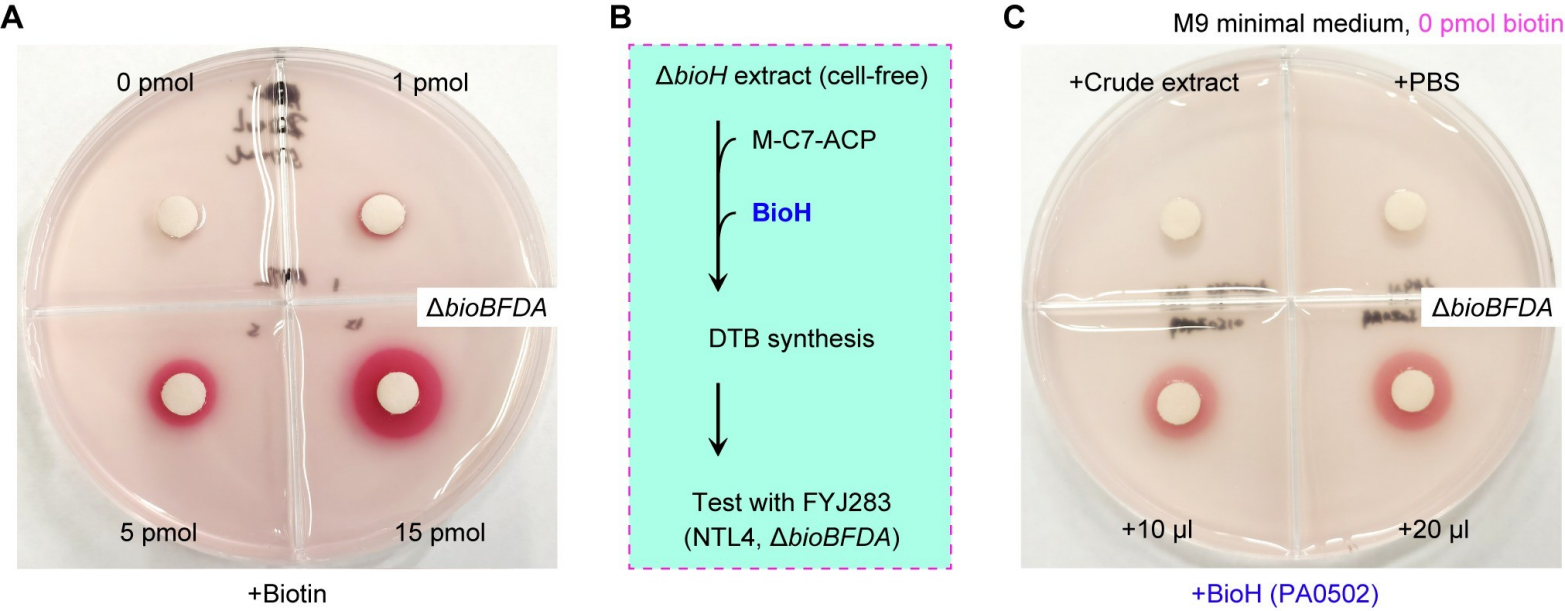
The BioH (PA0502) allows the *in vitro* reconstitution of biotin synthesis pathway. **A.** The addition of exogenous biotin allows growth of the biotin auxotrophic strain on non-permissive, biotin-deficient condition of M9 minimal medium. **B.** Scheme for the *in vitro* reconstitution of biotin synthesis. **C.** The PA0502 enzyme enables the reconstitution of biotin synthesis *in vitro*. The biotin indicator strain used here, is FYJ283 (the Δ*bioBFDA* mutant of *Agrobacterium tumefaciens* NTL4). Use of 2,3,5-triphenyl tetrazolium chloride (TTC, 0.001%) proceeds to probe bacterial viability of the Δ*bioBFDA* mutant. In principle, the occurrence of biotin is verified by the release of insoluble red pigment reduced from TTC that is precipitated to surround viable cells [17]. To prevent biotin cross-contamination, quadrant sectored plates were used, each sector of which is placed with a paper disc to spot biotin and/or DTB/biotin reaction mixture.

To visualize its metabolic role of *P*. *aeruginosa* BioH, we established the DTB/biotin bioassay coupled with the *in vitro* reconstituted system for DTB/biotin synthesis (**Fig 4A** and **4C**) as described by different research groups [17, 47, 62]. In this BioH-including system, the cell-free crude extract was prepared from the biotin auxotroph of *E*. *coli* Δ*bioH*, providing an array of enzymes of FASII and late steps of biotin synthesis (**Fig 4B**). The detection of DTB/biotin production relies on an indicator strain FYJ283, the Δ*bioBFDA* deletion mutant of *A*. *tumefaciens* we previously developed (**S2 Table**) [49, 59]. In principle, i) the indicator strain FYJ283 cannot grow on biotin-limiting M9 minimal agar plates, unless exogenous DTB/biotin is supplied; ii) bacterial growth of the reporter strain on such non-permissive condition demonstrates the generation of DTB/biotin in the reconstituted system *in vitro*; and iii) the biotin-dependent viability of an indicator strain reduces 2,3,5-triphenyl tetrazolium chloride (TTC), releasing an insoluble red pigment circle precipitated around alive cells. As expected, the indicator strain FYJ283 cannot be viable in the absence of exogenous biotin, but slightly grows in the presence of 1 pmol biotin (**Fig 4A**). It was noted that the supplementation of biotin (up to 5 pmol) can give robust bacterial growth (**Fig 4A**). As for the crude extract and PBS buffer used in the *in vitro* system, no contamination of trace DTB/biotin was sensitized with the indicator strain FYJ283, because of lacking an insoluble red pigment circle (**Fig 4C**). In contrast, the reaction mixture (10 μl)-arising DTB/biotin is enough to allow bacterial growth of the biotin auxotrophic strain, i.e., *A*. *tumefaciens* Δ*bioBFDA* mutant (**Fig 4C**). It is concluded that *P*. *aeruginosa* BioH enables the *in vitro* reconstitution of biotin synthesis.

### BioH is necessary for bacterial survival within macrophages

**Fig 5:**
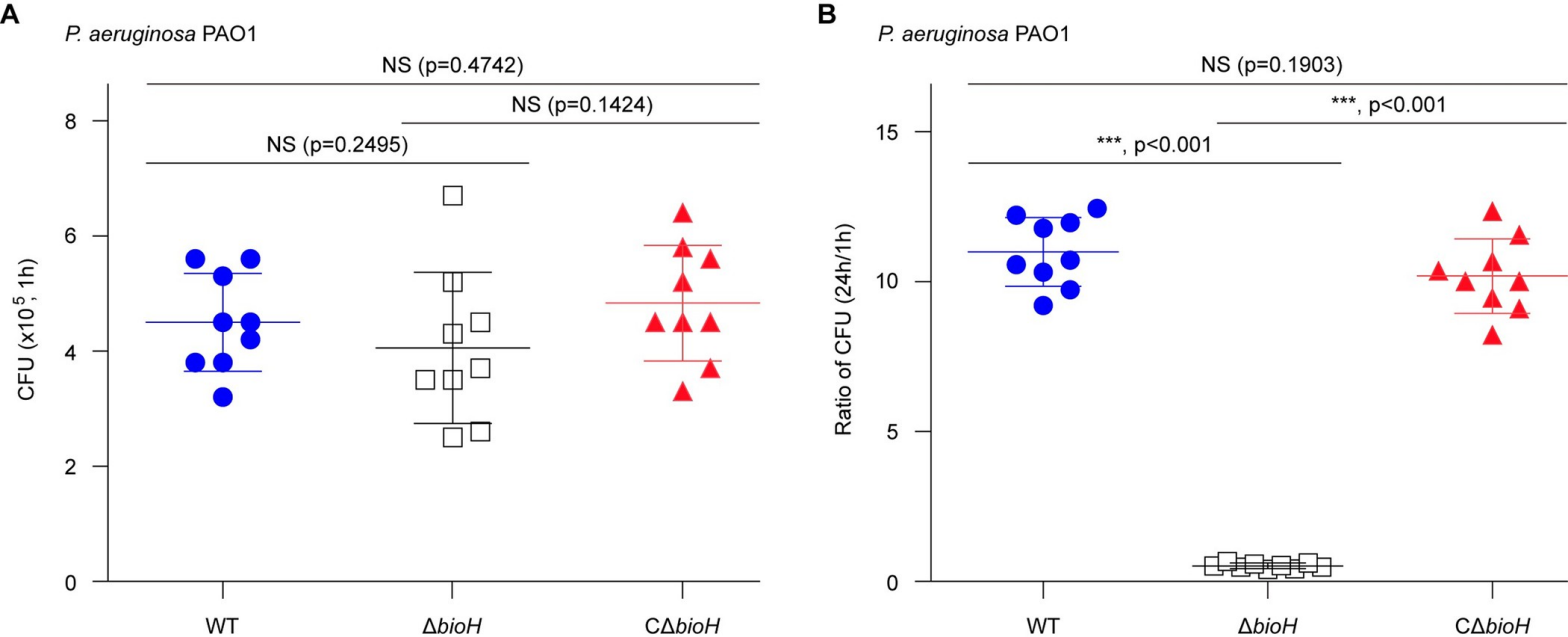
The requirement of BioH (PA0502) for survival of *P*. *aeruginosa* in mBMDM macrophage. **A.** Measurement of bacterial cell counts prior to challenge with *P*. *aeruginosa*. **B.** The removal of PA0502 dramatically impair the persistence of *P*. *aeruginosa* within macrophages. Data are shown as means ± SD, and were assayed by two-tailed analysis of variance. Designation: NS, not significant; WT, *P*. *aeruginosa* PAO1; Δ*bioH*, the ΔPA0502 mutant of *P*. *aeruginosa* PAO1; CΔ*bioH*, the complemented strain of the *P*. *aeruginosa* ΔPA0502 mutant with a plasmid pAK1900-borne PA0502.

In general agreement with the fact that the pathway of biotin synthesis and utilization participates in the intracellular pathogen *Francisella* infectivity [16–18], the BioA inhibitor MAC13772 was found to attenuate virulence of human pathogen *P*. *aeruginosa* by interfering the late step of biotin biosynthesis [15]. Thus, it is reasonable to speculate the involvement of *bioH* (PA0502) in the replication of *P*. *aeruginosa* within macrophages. To test this hypothesis, we carried out the analyses for macrophage infections. Murine bone marrow-derived macrophages (mBMDM) were prepared as Zhu *et al*. [63] described with little change. The prior exploration informed us that the appropriate value for multiplicity of infection (MOI) is estimated to be 5:1 (*P*. *aeruginosa* PAO1: mBMDM cell). In addition to the wild-type (WT) PAO1, the two derivatives of *P*. *aeruginosa* used to challenge mBMDM cells included i) the Δ*bioH* mutant, and ii) its complemented strain CΔ*bioH*. Here, 6 hours of co-cultivation ensured bacterial invasion into macrophages. After 1 h post-infection, we noted that the intra-phagosome persistence of Δ*bioH* is indistinguishable when compared with its parent type and the CΔ*bioH* strain (**Fig 5A**). This indicated no role of the *bioH* in immunological invasion of *P*. *aeruginosa* PAO1. However, the intracellularly-phagocytosed cells of the Δ*bioH* mutant are almost completely cleaned at 24 h post-infection. This was evidenced by the fact the ratio of CFU (24 h:1 h) is around 0.5 for the Δ*bioH* mutant, which is roughly 20-fold lower than that of the wild-type (**Fig 5B**). Furthermore, genetic complementation of the Δ*bioH* mutant restored its reproduction within mBMDM macrophage to the level of wild-type (**Fig 5B**). Thus, it is likely that *bioH* is critical for the replication of *P*. *aeruginosa* within macrophages.

### The repertoire of dynamic biotin levels within mouse tissues

**Fig 6:**
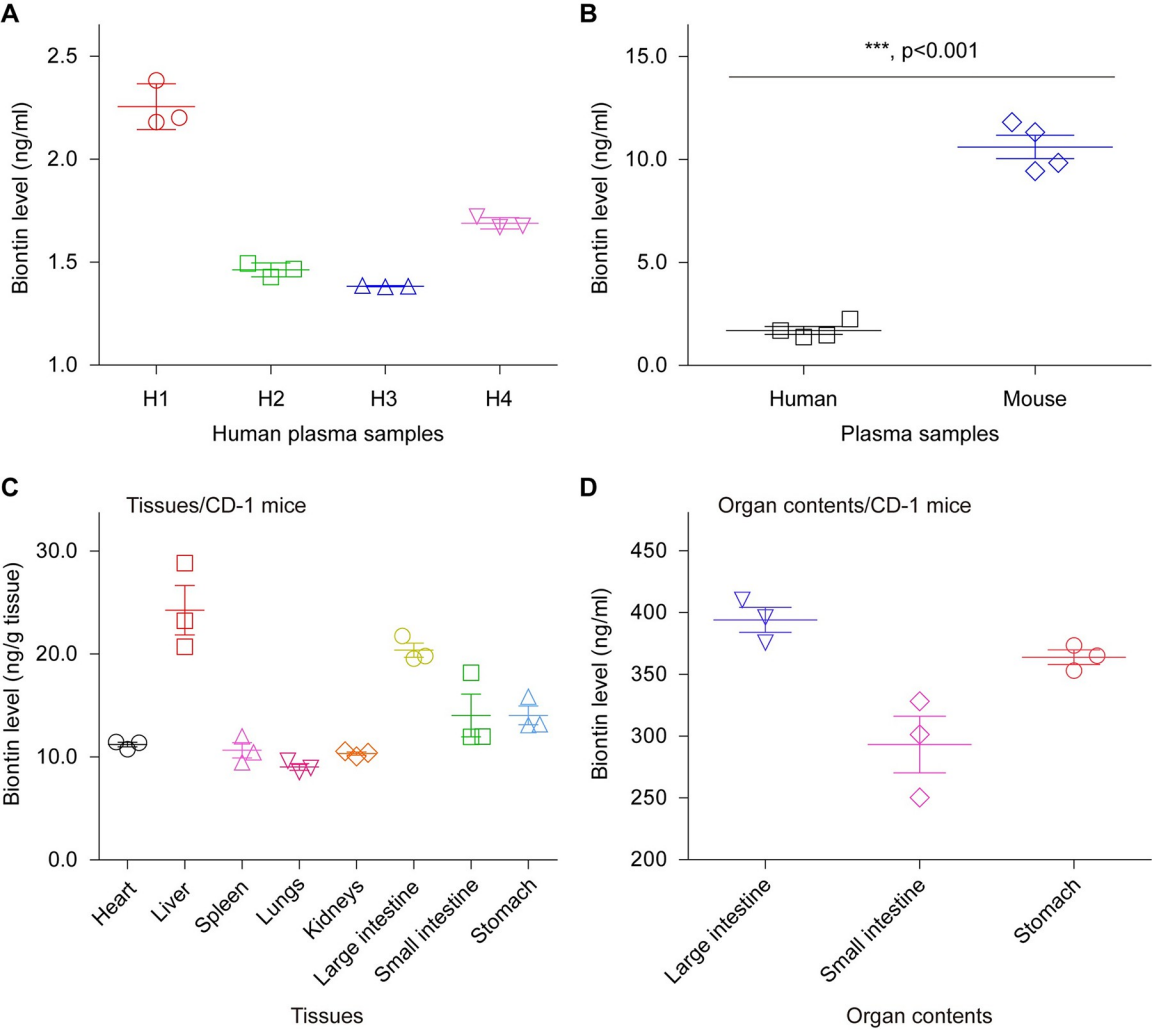
Comparison of biotin level in human plasmas and various mouse tissues (and/or organs). **A.** Relatively-low level of biotin is measured in human plasma samples. Blood plasmas of four human individuals (H1 to H4) were subjected for the routine biotin measurement. Biotin level of human plasma varies from 1.38 to 2.38 ng/ml (i.e., 5.65 nM to 9.74 nM), which is generally consistent with the observation with human biotin by Carfrae and coworkers [15]. **B.** Contrasting plasma biotin level in CD-1 mice and humans. The average biotin level (10.61 ng/ml; 43.43 nM) in mouse plasma is around 6-fold higher than the counterpart (1.70 ng/ml; 6.96 nM) in the human individual. **C.** The determined level of biotin assigned to an array of tissues of CD-1 mice Totally, eight tissues examined here include heart, liver, spleen, lungs, kidneys, large intestine, small intestine, and stomach. **D.** Measurement for biotin level in organ contents from CD-1 mice. Biotin level was measured using CD-1 mice (5–6 weeks). The organ contents were separately collected from large intestine, small intestine, and stomach. All the data are expressed in means ± SD, and assayed by two-tailed analysis of variance. Clearly, biotin level (300–400 ng/ml) in organ contents is over 20-fold higher than the equivalents (10–20 ng/ml) in associated tissues.

The recent finding of Carfrae *et al*. [15] informed us that biotin level in mouse plasma is estimated to be 40-fold higher than human plasma. As Carfrae and colleagues [15] conducted, we also employed an ELISA-based Biotin Quantitative Determination Kit to investigate the abundance of biotin pools in a panel of samples. First, we analyzed plasma biotin from four healthy individuals, and found its concentration varying from 1.38 to 2.38 ng/ml (equivalently 5.65 to 9.74 nM, **Fig 6A**). Second, the average biotin level in mouse plasma of CD-1 (5–6 week) lineage was measured to be 10.61 ng/ml (i.e., 43.43 nM), which is 6-fold higher compared to the counterpart (average value: 1.7 ng/ml; 6.96 nM) in human plasma (**Fig 6B**). This generally supported the proposal by Carfrae *et al*. [15] regarding the variation in plasma biotin levels. Whereas we believed that the discrepancy in fold change is mainly due to the difference on human plasma sources used in the two studies. Third, we quantified biotin levels of eight mouse tissues, namely heart, liver, spleen, lungs, kidneys, large intestine, small intestine, and stomach (**Fig 6C**). Except that both liver and large intestine display relatively-higher biotin level (~20 ng/g tissue, **Fig 6C**), all the remaining 6 tissues possess biotin at close levels of ~10 ng/g tissue, which is similar to that of mouse plasma (**Fig 6B** and **6C**). Finally, we probed biotin abundance in three kinds of mouse organ contents, which separately refer to large intestine, small intestine, and stomach. Intriguingly, biotin levels in these organ contents (300 to 400 ng/ml) were observed to be approximate 20-fold higher than those of the corresponding organs (**Fig 6C** and **6D**). When compared to mouse plasma, they contain biotin at nearly 30 to 40-fold higher levels (**Fig 6B** and **6D**). This can be explained partially by the fact that they are places where a large amount of biotin produced by the gut microbiota are secreted to feed certain biotin-consuming residents [44, 64].

**Fig 7:**
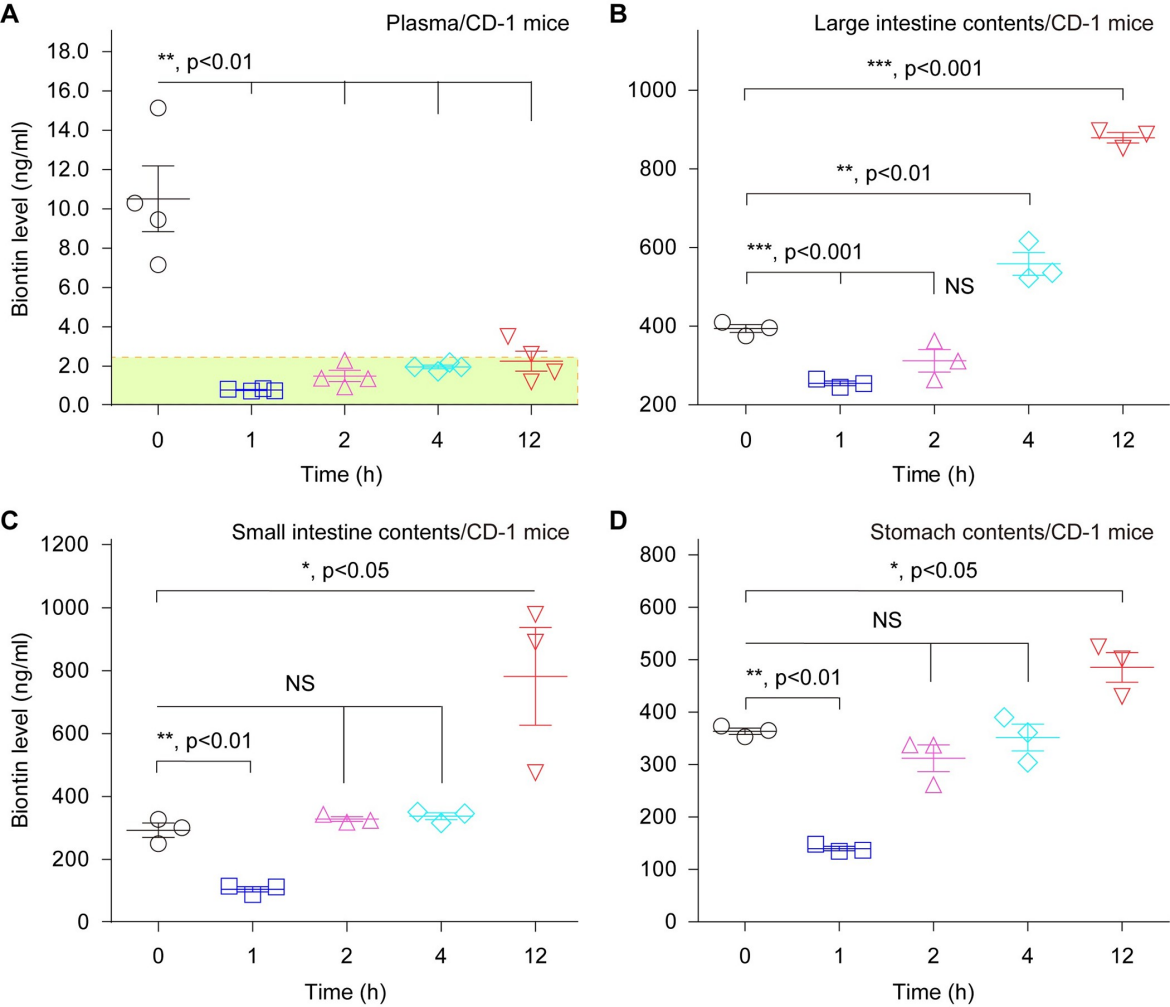
Intraperitoneal administration with streptavidin benefits an establishment of a CD-1 mice model for *P*. *aeruginosa*, of which the plasma (and organ contents) biotin levels are dramatically decreased at an initial infection stage. **A.** Intraperitoneal administration of CD-1 mice with streptavidin renders its plasma biotin decreased at the comparable level with human plasmas. The range of human plasma biotin level (1.38 to 2.38 ng/ml) is indicated with a rectangle shadowed green. The biotin levels in large intestine contents (**B**), small intestine contents (**C**), and stomach contents (**D**), are reduced significantly at 1-hour post-intraperitoneal administration with streptavidin. To mimic human infection with *P*. *aeruginosa*, the model of CD-1 mice is subjected for an intraperitoneal administration with streptavidin (2 mg/kg) as recently Carfrae *et al*. [15] recommended, which is supposed to reduce the biotin levels in mouse plasma and other organ contents. All the data are given in means ± SD, and assayed by two-tailed analysis of variance.

The use of an experimental model mimicking human biotin levels is a prerequisite for evaluating a role of *bioH* in *P*. *aeruginosa* infection. As recommended by Carfrae and coworkers [15], CD-1 mice were intraperitoneally administered with streptavidin (~2 mg/kg). Dynamic level of mouse biotin *in vivo* was monitored during the 12 h period post-administration with streptavidin. As expected, the mouse plasma biotin concentration remains at low level of less than 2 ng/ml, comparable to human plasma (**Fig 7A**). In particular, the lowest level of plasma biotin that is 0.79 ng/ml (3.23 nM), occurs at 1 h post-administration with streptavidin (**Fig 7A**). Similar scenarios were observed with the contents arising from the bellowed organs, namely large intestine (**Fig 7B**), small intestine (**Fig 7C**), and stomach (**Fig 7D**). Also, the biotin concentrations dramatically dropped at 1 h after an intraperitoneal administration with streptavidin, regardless of its elevation over time (**Fig 7A**–7**D**). The landscape of biotin dynamic alteration was reproduced in the eight mouse tissues we examined (**S4 Fig**), which separately included heart (**S4A Fig**), liver (**S4B Fig**), spleen (**S4C Fig**), lungs (**S4D Fig**), kidneys (**S4E Fig**), large intestine (**S4F Fig**), small intestine (**S4G Fig**), and stomach (**S4H Fig**). These data combined with observations of Carfrae *et al*. [15] revealed that the challenge of CD-1 mice with *P*. *aeruginosa* at 1 h after the pre-administration with streptavidin (2 mg/kg) is supposed to mostly mimic human infection of with *P*. *aeruginosa*. In summary, we presented a repertoire of dynamic biotin levels in various organs/tissues of CD-1 mice. Along with findings of other research groups, this observation might benefit understanding multi-faceted roles of biotin in bacterial infection [15], and pathophysiology of mammalian animals [44, 64].

### Removal of *bioH* attenuates the pathogenicity of *P*. *aeruginosa* in a murine model of systemic infection

**Fig 8:**
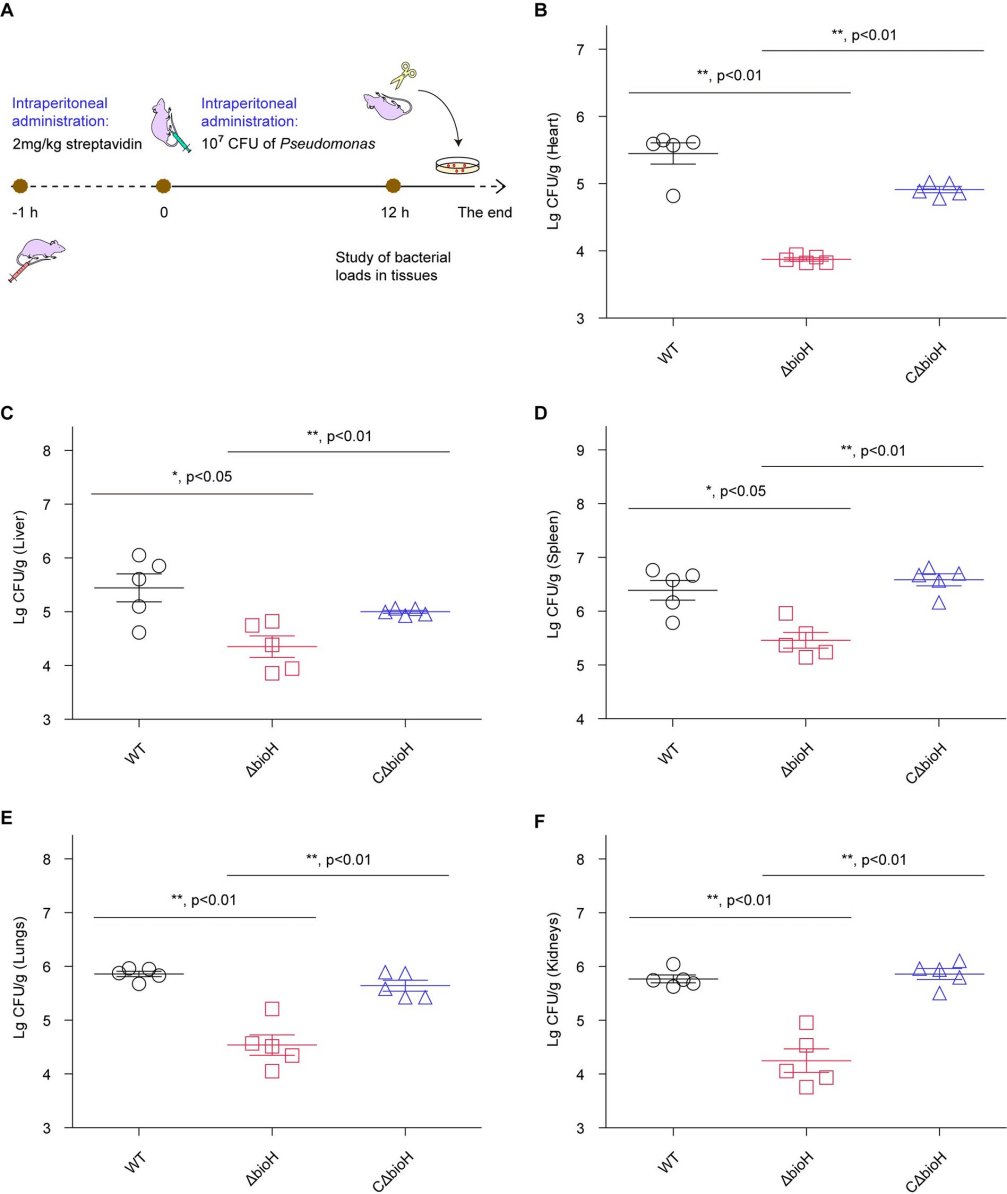
Intraperitoneal challenge suggested that the removal of *bioH* impairs its persistence of *P*. *aeruginosa* within CD-1 mice pre-administered with streptavidin. **A.** Scheme for a model of CD-1 mice intraperitoneally challenged with *P*. *aeruginosa* derivatives. The CD-1 (5–6 weeks) mice are pre-administered with streptavidin, at 1h prior to the challenge with *P*. *aeruginosa*. Of note, the cartoon models were drawn by ourselves, on the basis of certain templates freely provided by ScienceSlides Online. **B.** Significant reduction in bacterial loads of the Δ*bioH* mutant of *P*. *aeruginosa* in the heart of the infected CD-1 mice. The impaired survival of the *P*. *aeruginosa* Δ*bioH* mutant within a series of tissues of the infected mouse, namely liver (**C**), spleen (**D**), lungs (**E**), and kidneys (**F**). The re-introduction of a plasmid-borne *bioH* into the Δ*bioH* mutant restores its abilities of replicating in the aforementioned tissues. All the data are expressed in means ± SD, and checked with two-tailed analysis of variance. Designations: WT, the wild-type strain of *P*. *aeruginosa* PAO1; Δ*bioH*, the mutant of *P*. *aeruginosa* devoid of *bioH*; CΔ*bioH*, the genetically-complemented strain of Δ*bioH* mutant.

The experimental model for systemic infections we established here denotes an intraperitoneal challenge of CD-1 mice with 1x 10^7^ CFU of *P*. *aeruginosa*, in which i) a pre-administration with 2 mg/kg of streptavidin proceeds one hour earlier, and ii) bacterial loads are determined by plating the dilution of homogenized tissues from the mouse euthanized 12 h post-infection (**Fig 8A**). In addition to the WT *P*. *aeruginosa* PAO1 strain as a positive control of strong invasiveness, the mutant strain of Δ*bioH* was evaluated, as well as its genetically-complemented strain, CΔ*bioH* (**S2 Table**). Here, five different tissues/organs were sampled for the analyses of bacterial loads, namely heart (**Fig 8B**), liver (**Fig 8C**), spleen (**Fig 8D**), lungs (**Fig 8E**), and kidneys (**Fig 8F**). The bacterial loads of the Δ*bioH* mutant in all examined tissues declined greatly in comparison with its parental strain, and such a growth defect within host can be largely restored by genetic complementation of a plasmid-borne *bioH* (**Fig 8B**–**8F**). We also examined bacterial loads in the routine model of CD-1 mice without the pre-administration with streptavidin (**S5A Fig**). As predicted, removal of *bioH* failed to exert any obvious effect on bacterial burden in the infected mouse tissues, including heart (**S5B Fig**), liver (**S5C Fig**), spleen (**S5D Fig**), lungs (**S5E Fig**), and kidneys (**S5F Fig**). It demonstrated that we have success in the establishment of systemic infection model of CD-1 mice mimicking the human environment, initially proposed by Carfrae and colleagues [15]. Importantly, the data indicated that the BioH gatekeeper links bacterial biotin synthesis to *P*. *aeruginosa* persistence within CD-1 mouse that mimics human plasma biotin levels.

### Deletion of *bioH* reduces the virulence of *P*. *aeruginosa* in mouse lung infections

**Fig 9:**
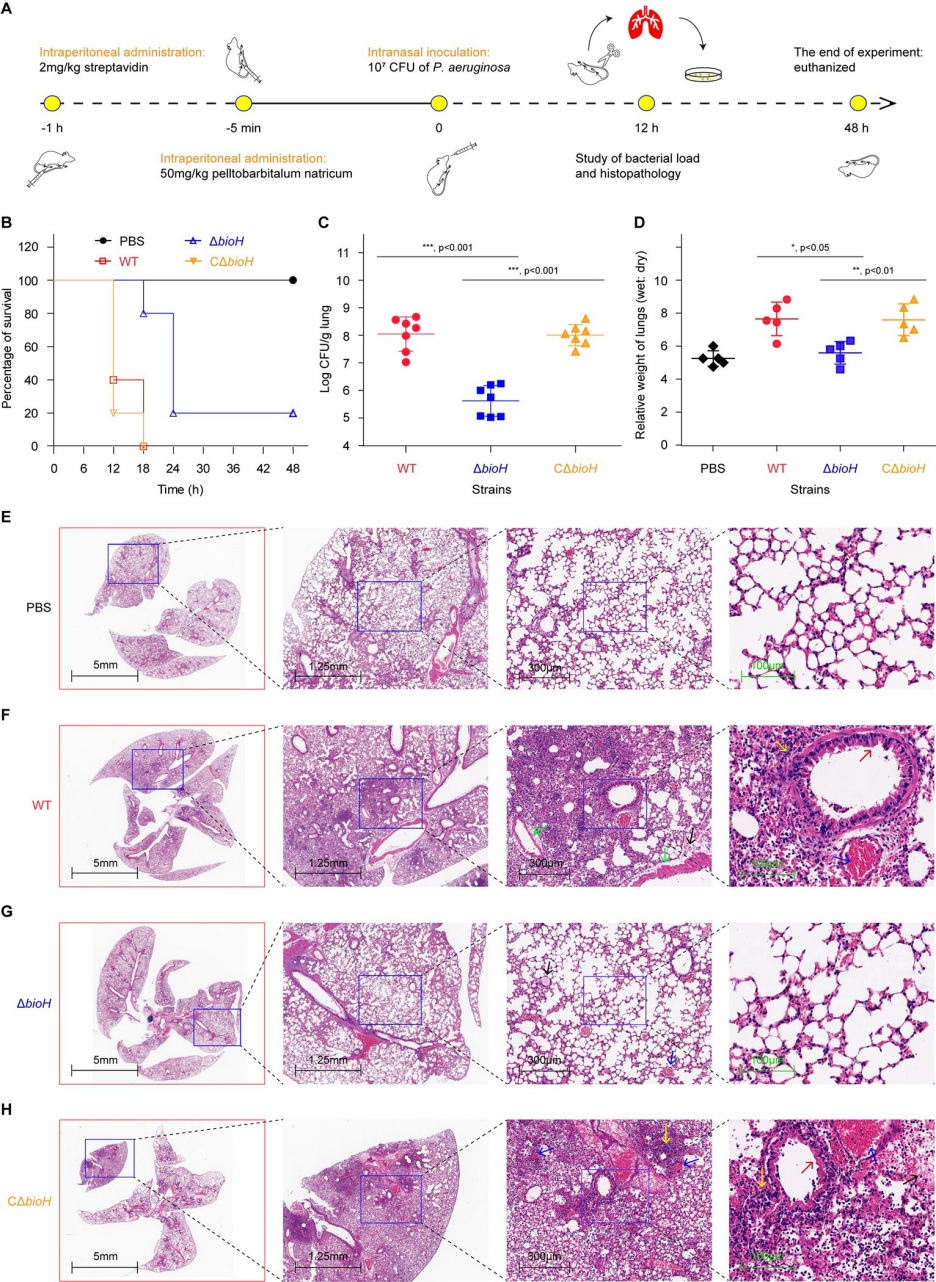
Intranasal infections of CD-1 mice elucidated that BioH (PA0502) plays a role in *P*. *aeruginosa* virulence. **A.** A strategy designed for mice challenge with *P*. *aeruginosa* via an intranasal administration. As described in Fig 8A, the cartoon models were generated by ourselves. **B.** Survival curves of CD-1 mice infected with various strains of *P*. *aeruginosa*. **C.** Bacterial load of the lung tissues from mice post-infection with three types of *P*. *aeruginosa* strains. **D.** Relative ratio (wet weight: dry weight) of the lung tissues from mice post-infection with three types of *P*. *aeruginosa* strains. Data are shown as means ± SD, and were analyzed in two-tailed way of variance via Mann Whitney test. Representative histopathology of the lung tissue from mice inoculated with the blank control PBS (**E**) and the positive control, wild-type strain PAO1 of *P*. *aeruginosa* (**F**). **G.** Pathological section of the lung tissue from mice infected with the Δ*bioH* mutant of *P*. *aeruginosa* PAO1. **H.** Histopathological analyses of the lung tissues collected from mice challenged with the complemented strain (CΔ*bioH*) of the *P*. *aeruginosa* Δ*bioH* mutant. The lung tissues were collected from infected and placebo mice, which were killed and necropsied on 2 dpi. Lung samples were stored in formalin for 7 days and subsequently embedded in paraffin. Finally, hematoxylin and eosin were applied in staining of sectioned samples. PBS denotes the blank control group, and WT is the positive control group. Scale bars are labeled on each panel. In the two groups of mice infected with WT (**F**) and CΔ*bioH* (**H**), lung lesions displayed i) thicken alveolar septa (red arrows); ii) multifocal hemorrhage in certain alveolar cavities (blue arrows) and bronchiolar cavities (green arrows); iii) homogeneously pink infiltration in partial alveolar cavities (black arrows); and iv) inflammatory cell accumulation in certain alveolar spaces and cavities [including lymphocytes, macrophages and neutrophils (yellow arrows)]. Designation: WT, *P*. *aeruginosa* PAO1; Δ*bioH*, the ΔPA0502 mutant of *P*. *aeruginosa* PAO1; CΔ*bioH*, the complemented strain of the *P*. *aeruginosa* ΔPA0502 mutant with a plasmid pAK1900-borne PA0502.

To further determine the role of *bioH* in *P*. *aeruginosa* virulence, we adopted a mouse lung infection model. In light that the abundance of biotin is 6-fold (40-fold in the measurement of Carfrae *et al*. [15]) higher in mouse plasma compared to human plasma (**Fig 6B**), we also leveraged streptavidin to mimic human biotin environment in CD-1 mice prior to lung infections (**Fig 9A**). Apart from the negative control group inoculated with PBS buffer, the remaining three groups of mice (10/group) were separately challenged with WT*/*PAO1, Δ*bioH*, and CΔ*bioH*. Unlike the negative group of which all the 10 mice are not sacrificed within the whole monitoring period of 48 hours (**Fig 9A** and **9B**), none of mice from the group injected with wild-type PAO1 (and/or the complemented strain CΔ*bioH*) survived at 18 h post-infection (**Fig 9B**). The survival ratio of the group challenged with Δ*bioH* was 80% (8/10) at 18 h post-infection, and two mice remained alive by the end of infection (**Fig 9A** and **9B**). This suggested that the *bioH* plays a modest role in *P*. *aeruginosa* pathogenicity with CD-1 mouse model. The differences in the CFUs (substantial) versus survival curves (not substantial) could be a reflection of this point, where *P*. *aeruginosa* initially can be largely cleared in the host “nutrient adjustment period”, but might eventually scavenge/compete host biotin to maintain an infection.

Next, we settled out to evaluate pathological roles through measuring i) bacterial load in lungs and ii) pulmonary edema at 12h post-infection. First, we found that the level of Δ*bioH* collected from lungs is over 2-log lower than the wild-type PAO1 (**Fig 9C**). As expected, the replication defect of Δ*bioH* in lungs was significantly reversed upon the introduction of a plasmid-borne PA0502 (**Fig 9C**). The symptom of pulmonary edema was routinely approached with the ratio of wet weight of lung to its dry weight. As for the lungs infected with wild-type PAO1, the ratio was calculated to be about 8, much higher than the equivalent value (~5) of negative control group inoculated with PBS buffer (**Fig 9D**). Whereas the Δ*bioH*-infecting group gave the count of ~5, quite close to that of the PBS group. In the group challenged with the CΔ*bioH* strain, the ratio was restored to the level of the PAO1 strain (**Fig 9D**). Clearly, the inactivation of *bioH* potently compromised pulmonary edema, a hallmark of severe lung injuries.

Using histopathological analyses of lungs, we observed that in contrast to the negative control (i.e., PBS group) that displays an intact alveolar structure (**Fig 9E**), the wild-type PAO1-injecting mice exhibited intensive lung symptoms featuring with thicken alveolar septa and inflammatory cell accumulation in certain regions (**Fig 9F**). Not surprisingly, the lung section arising from the group infected with Δ*bioH* mutant presented almost intact alveolar structure except for particular multifocal hemorrhage and homogeneously pink infiltration (**Fig 9G**). Of note, the re-introduction of plasmid-borne PA0502 expression also caused similar pathological lesions as the parental strain PAO1 does (**Fig 9H**). The combined data allowed us to conclude that BioH (PA0502) is important, if not essential, for lung infections of mice with *P*. *aeruginosa* PAO1.

### *Pseudomonas* biotin synthesis is a druggable pathway

**Fig 10:**
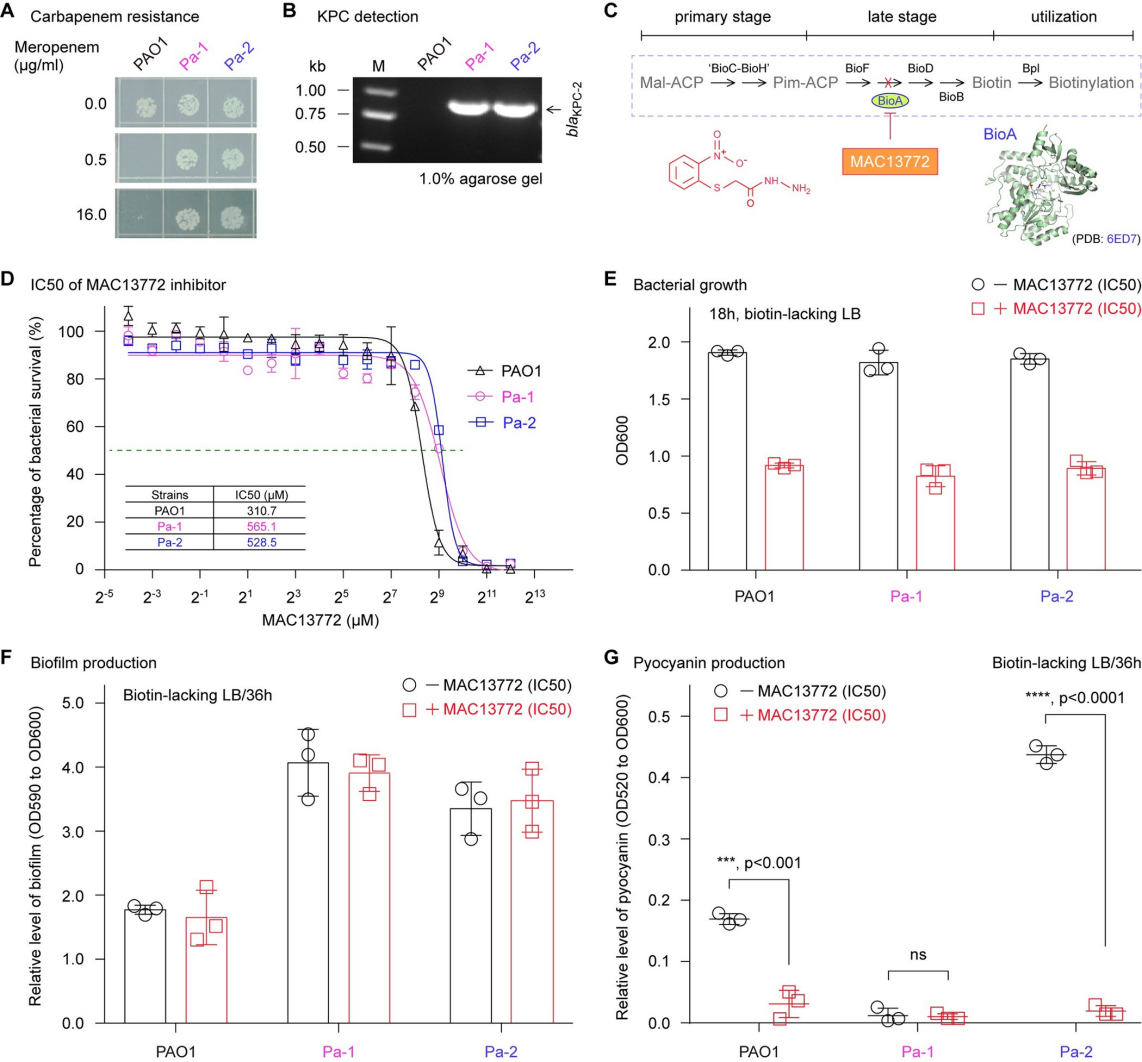
The biotin synthesis inhibitor MAC13772 dampens CRPA viabilities, and selectively interferes the formation of an important virulence factor pyocyanin. Resistance analysis (**A**) and molecular assays (**B**) of two CRPA isolates Pa-1 and Pa-2. Both Pa-1 and Pa-2 harbor *bla*_KPC-2_. Unlike Pa-2 producing the secreted virulence factor pyocyanin, the clinical isolate Pa-1 is a natural mutant defective in pyocyanin synthesis. **C.** Scheme for MAC13772 known as an inhibitor of BioA, an important enzyme responsible for late stage of *Pseudomonas* biotin synthesis. In addition to crystal structure of BioA (PDB: 6ED7), chemical structure of BioA inhibitor, MAC13772 was given [15]. **D.** Measurement for IC50 of MAC13772 in the viabilities of two CRPA isolates and PAO1 virulent strain. **E.** 18h-growth biomass of three *P*. aeruginosa in the biotin-lacking LB stressed with IC50 of MAC13772. **F.** No effect on the virulence factor biofilm production exerted by MAC13772 in the biotin-lacking LB growth condition. **G.** Evidence that MAC13772 can interfere profoundly synthesis of the virulence factor pyocyanin in *P*. *aeruginosa* isolates.

We next assessed the vulnerability of *P*. *aeruginosa* by intervening biotin metabolism. In addition to the representative virulent strain, *P*. *aeruginosa* PAO1, we also included two clinical CRPA isolates (Pa-1 and Pa-2, in **Fig 10A**) in the assays. The insusceptibility to meropenem was attributed to the existence of *bla*_KPC-2_ (**Fig 10B**). Unlike the two strains PAO1 and Pa-2, Pa-1 seemed to be a natural mutant defective in pyocyanin production. Since MAC13772 is known to efficiently inhibit the activity of BioA, an important enzyme responsible for late step of biotin synthesis (**Fig 10C**) [65], we evaluated bacterial viabilities on the cultivation conditions containing varied levels of MAC13772 inhibitor. In general consistency with the description of Carfrae *et al*. [15], we observed that all the three examined strains of *P*. *aeruginosa* are inhibited greatly, regardless of carbapenem resistance (**Fig 10D**). The half-maximal inhibitory concentrations (IC50) of MAC13772 are 310.7 μM for PAO1, 565.1 μM for Pa-1, and 528.5 μM for Pa-2, respectively (**Fig 10D**). Similarly, the reduction in bacterial growth was also seen with LB medium treated with streptavidin to minimize the availability of free biotin (**Fig 10E**). As anticipated, the biotin-eliminated LB medium enables the production of two *Pseudomonas* virulence factors [i.e., biofilm [66, 67] and pyocyanin [68, 69]]. On such condition, we thereafter investigated the MAC13772-stressed formation of biofilms and pyocyanin. Although the presence of MAC13772 inhibitor had no obvious role in modulating formation of *Pseudomonas* biofilms (**Fig 10F**), it significantly attenuated production of the other virulence factor pyocyanin (**Fig 10G**). Thus, we concluded that *Pseudomonas* biotin synthesis can be a druggable metabolic pathway.

## Conclusions

Prior to this study, bacterial biotin metabolism has already been connected with successful infections of two notorious intracellular pathogens, namely i) the TB-causing bacterium, *M*. *tuberculosis* [40, 70]; and ii) the life-threatening agent, *F*. *tularensis* [16–18]. The data we reported here represents a first proof that BioH, an important member of the toolbox of biotin synthesis enzymes, is critical for the pathogenesis of *P*. *aeruginosa*, an extracellular pathogen (**Figs 5**, **8** and **9**). The prototypical C7-ACP methyl ester demethylase BioH functions as a gatekeeping enzyme in the canonical biotin synthesis pathway, because that it determines the switching from the modified FASII cycle to the late step of biotin biosynthesis (**Fig 1B**). Notably, the BioH of KPC-2-producing clinical *P*. *aeruginosa* with carbapenem resistance is identical to the counterpart of the model *P*. *aeruginosa* PAO1. Although that its expression and activity is independently of genetic context [56], removal of *bioH* renders *P*. *aeruginosa* PAO1 biotin auxotrophic (**Figs 1** and **2**). In spite that genomic environment of ‘BioC-BioH’ path markedly varies in diverse microbes, BioH is paired with BioC at the constant ratio of 1:1. The advantage of this genetic arrangement is to prevent wasteful production of two enzymes dedicated to the primary stage of biotin synthesis. Whereas a rare example of exception was recently discovered in *Mycobacterium smegmatis*, i.e., three BioH isoforms (BioH1 to BioH3) combined with a single BioC, are programed into an early stage of mycobacterial biotin synthesis [62]. Presumably, the redundance of BioH-like activities is originated from the domestication of certain α/β-hydrolase to gain this promiscuous role of hydrolyzing M-C7-ACP ester. The evolutionary advantage of redundant BioH isoenzymes for mycobacterial pathogens is to guarantee efficient biotin production within harsh host niche. This is because that the BioH action proceeds smoothly even one or two isoenzymes are impaired or hijacked by host immunity to do another job.

To the best of our knowledge, Carfrae *et al*. shed light on the previously-unknown human plasma environment, of which biotin is unexpectedly 40-fold lower than that of mouse model involved in our routine infection study [15]. In contrast, we found that plasma biotin concentration of CD-1 mice is around 6-fold higher than that of human plasma (**Fig 6A** and **6B**). The discrepancy is probably due to the altered origin of human plasmas. Nonetheless, these data benefited the establishment for infection model of mouse mimicking human niches. In particular, we presented a repertoire of distinct biotin levels in various mouse tissues and organ contents, which is helpful to understand interplay between *P*. *aeruginosa* and host in views of competing for metabolic nutrients/cofactors. Similar to those of plant pathogen *A*. *tumefaciens* [59] and human pathogen *M*. *tuberculosis* [61], *P*. *aeruginosa* encodes up to 5 biotin-requiring enzymes (**S1 Table** and **Fig 2B–2G**). This explains in part, if not all, why the physiological requirement of biotin in *P*. *aeruginosa* is 10 to 20-fold higher than that of *E*. *coli* having a single biotinylated protein, AccB (**Fig 2A**–**2C**). Like the paradigm *E*. *coli* BioH [47] and its unrelated isoenzyme BioJ [17], *P*. *aeruginosa* BioH can enable the *in vitro* reconstitution of biotin synthesis (**Fig 4**), verifying its biochemical role. These observations highlight the importance of biotin cofactor in central metabolism and pathophysiology of the opportunistic agent, *P*. *aeruginosa*. As for *Francisella*, the category A tier 1 select agent, the gatekeeper enzyme BioH is well-known to play a role in survival and immunity escape from macrophages [17, 18]. Different from *Francisella* that is a facultative extracellular bacterium, *P*. *aeruginosa* is an obligate extracellular pathogen that causes opportunistic human infections. The finding that *P*. *aeruginosa* BioH participates into bacterial infections allowed us to believe that *de novo* biotin synthesis might benefit intramacrophage persistence rather than phagosome-inside replication (**Fig 5A**). Given the promiscuity of BioH demethylase, it can’t rule out the possibility that certain non-specific substrate selectivity other than biotin synthesis might be involved in *P*. *aeruginosa* pathogenesis. Whereas alternative mechanisms remain to be defined in the near future.

The catalytic triad (S82, D207 & H235 for *E*. *coli* BioH) is ubiquitously present amongst diverse BioH orthologs (**Figs S1** and **3A**) and non-homologous isoforms, exemplified with *Francisella* BioJ [17] and *Helicobacter* BioV [55]. Apart from the contribution to its enzymatic activity, the two residues (S66 and D189) of *P*. *aeruginosa* BioH catalytic triad are unexpectedly found to be critical for the maintenance of its solution structure (**S3A Fig**). Thus, it is of much interest to test if a similar scenario is seen with the paradigm *E*. *coli* BioH and its non-closely relative BioJ. Apart from biotin synthesis route, the machinery of biotin utilization is also implicated into bacterial pathogenicity [16, 71]. It was noted that the biotin protein ligase, Bpl appears to be an alternative front-line anti-TB drug target [40]. The extracellular pathogen *P*. *aeruginosa* possesses a bifunctional Bpl/BirA that is also a repressor for biotin synthesis. It is reasonable to ask the question if *P*. *aeruginosa* Bpl/BirA is a restricted/nutritional virulence factor. In this work, we also approved that the known BioA inhibitor MAC13772 are active with both PAO1 and clinical CRPA isolates (**Fig 10**). More importantly, this inhibitor profoundly interferes formation of a well-known virulence factor pyocyanin, regardless of KPC-2 carbapenem resistance (**Fig 10B** and **10G**). Notably, our epidemiological screen returned a natural mutant (designated as Pa-1) of *P*. *aeruginosa* that is defective in pyocyanin synthesis (**Fig 10A** and **10G**). However, the underlying mechanism awaits further exploration in the near future.

Collectively, genetic and infection studies of *P*. *aeruginosa* BioH posed a link between intra-macrophagic biotin and rapid phagosomal persistence/escape. The success of bacterial biotin metabolism in clinical anti-TB translation trials [43], prompted us to believe BioH-including enzyme toolbox as a promising drug target that can re-sensitize CRPA in the development of lead compounds and front-line antibiotics.

## Materials and methods

### Ethics statement

The maintenance and manipulation of the opportunistic pathogen *Pseudomonas aeruginosa* was carried out in the biosafety level 2 (BSL-2) laboratories from Zhejiang University School of Medicine, and Shanghai Institute of Materia Medica, Chinese Academy of Sciences. The analyses of mice infections were conducted under the relevant guidelines and regulations from the Administration of Affairs Concerning Experimental Animals approved by the State Council of People’s Republic of China (11-14-1988). The protocols of animal study were reviewed and approved by the Institutional Animal Care and Use Committee (IACUC) of the Shanghai Public Health Clinical Center (permit 2013P201). The laboratory animal usage license numbers included SYXK-HU-2010-0098 (certified by Shanghai Committee of Science and Technology, and ZJU20220126 (licensed by Laboratory Animal Welfare and Ethics Committee of Zhejiang University). Under appropriate regulation, four samples of healthy human plasma were kindly provided by Clinical Laboratory of the 2^nd^ Affiliated Hospital (School Hospital Branch), Zhejiang University School of Medicine.

### Bacterial strains and growth conditions

Two types of bacterial strains used in this study arose from *Escherichia coli* (*E*. *coli*) MG1655 and *Pseudomonas aeruginosa* (*P*. *aeruginosa*) PAO1, and maintained at 37°C. Strains DH5α and BL21 (DE3) acted as prokaryotic hosts for gene cloning and protein expression, respectively (**S2 Table**). STL24 (MG1655, Δ*bioH*), the biotin auxotroph functioned as a recipient host to test the function of *bioH* (PA0502) and its 3 point-mutants (**S2 Table**). Strain FYJ5201, the *bioH* (PA0502) deletion mutant (Δ*bioH*) of *P*. *aeruginosa* PAO1, were evaluated on non-permissive condition of biotin-lacking M9 minimal medium, as well as its genetically-complemented strain, FYJ5202 (CΔ*bioH*) carrying the plasmid-borne *bioH* (PA0502), pAK1900::*bioH* (**S2** and **S3 Tables**). Totally, five engineered strains (FYJ5214 to FYJ5216, FYJ5226, and FYJ5228) were created to prepare the putative biotinylated enzymes of PAO1 (**S2 Table**). In addition to Luria-Bertani (LB) broth, the chemicals-defined M9 minimal medium with or without biotin was utilized. When necessary, antibiotics were supplemented as follows: 100 μg/ml for ampicillin, 100 μg/ml for carbenicillin, and 50 μg/ml for kanamycin.

### Antibacterial analyses of BioA inhibitor, MAC13772

The known BioA inhibitor MAC13772 was also used to approve the feasibility of *Pseudomonas* biotin biosynthesis as a promising druggable target [65]. In addition to the wild-type strain PAO1, two clinical *P*. *aeruginosa* isolates with carbapenem resistance were explored for the susceptibility to MAC13772. To figure out the resistance determinant, PCR assays were performed with a pair of *bla*_KPC-2_-specific primers. As Carfrae and coworkers described [15], all the three strains (PAO1, Pa-1, and Pa-2) were grown in biotin-free M9 minimal medium supplemented with MAC13772 in series of dilution. As a result, the plotting of growth curves gave the half-maximal inhibitory concentration (IC50) of MAC13772. Following the treatment with Streptavidin [15], the biotin-lacking LB medium was given. The resultant LB medium mixed with MAC13772 inhibitor at the IC50 level, acted as ‘selective medium’ for production of two virulence factors (i.e., biofilms and pyocyanin) in different *Pseudomonas* strains. The biofilms produced from static cultures, were stained with the dye crystal violet (0.1%), rinsed with water, and then quantified with a plate reader at one of the following 3 wave-lengths (550 nm, 570 nm or 590 nm) [66, 67]. The pyocyanin secreted into medium, were routinely extracted with chloroform solution, and measured in a plate reader at 520 nm [68, 69]. Finally, the value of biofilm/pyocyanin production is determined by calculating the ratio of OD590 (or OD520) to bacterial cell density (OD600).

### Genetic inactivation and complementation of PA0502 in *P*. *aeruginosa*

The sucrose counterselection against levansucrase (SacB)-aided approach was employed to inactive the *bioH* (PA0502) in the strain PAO1 of *P*. *aeruginosa*. The two adjacent regions (~1kb) of PA0502 were PCR amplified with 2 sets of specific primers (PA0502-up-F/R plus PA0502-down-F/R), and then fused together through overlapping PCR with the pair of primers (PA0502-up-F/PA0502-down-R, **S3 Table**). The resultant overlapped DNA fragment of around 2kb was directly cloned into the gene replacement vector pEX18Ap via two cuts of EcoRI and HindIII [72, 73], yielding pEX18Ap::PA0502-UD (**S2 Table**). Subsequently, the gentamicin resistance cassette (~1.8 kb) cut from pPS858 with BamHI, was inserted into pEX18Ap::PA0502-UD, giving the knock-out plasmid of pEX18Ap::PA0502-UGD (**S2 Table**). The resultant plasmid of pEX18Ap::PA0502-UGD was electroporated into PAO1 competent cells with the selection for gentamicin resistance. Colonies of interest were screened for gentamicin sensitivity and loss of sucrose (5%) sensitivity, which typically indicates a double cross-over event and thus marks the occurrence of gene replacement. The PA0502 deletion mutant of PAO1, designated FYJ5201, was verified with multiplex PCR and direct sequencing of the PCR amplicon. Additionally, the *bioH* (PA0502)-coding sequence was introduced into pAK1900 vector via HindIII/XbaI cuts, generating a complementary plasmid pAK1900::*bioH* (**S2 Table**). Finally, the resultant plasmid was electroporated into FYJ5201 (PAO1, Δ*bioH*), producing a genetically-complemented strain FYJ5202 that expresses a plasmid-borne PA0502, pAK1900::*bioH* (**S2 Table**).

### Molecular techniques and plasmids

The predictive *bioH* gene (PA0502) were amplified with PCR from *P*. *aeruginosa* PAO1 (and/or a CRPA isolate), and cloned into three types of vectors via homologous recombination. As a result, it separately gave pET28a::*bioH*, pBAD24::*bioH*, and pAK1900::*bioH* (**S3 Table**). Site-directed mutagenesis method was applied to further generate a series of BioH (PA0502) mutants defective in the catalytic triad (S66, D189 and H216). Namely, they included 3 pET28a derivatives [pET28a::*bioH*(S66A), pET28a::*bioH*(D189A) & pET28a::*bioH*(H216A)]; 3 pBAD24-based constructs [pBAD24::*bioH*(S66A), pBAD24::*bioH*(D189A) & pBAD24::*bioH*(H216A)]; and 3 pAK1900-borne mutants [pAK1900::*bioH*(S66A), pAK1900::*bioH*(D189A) & pAK1900::*bioH*(H216A)] (**S3 Table**). Five candidate genes that encode biotinylated enzymes (PA1400, PA2012, PA2891, PA4847, and PA5435) were engineered into pET21a (and/or pET28a), producing the recombinant plasmids as follows: pET21a::PA1400, pET21a::PA2012, pET21a::PA2891, pET28a::PA4847, and pET28a::PA5435 (**S3 Table**). All the plasmid constructs were confirmed with direct DNA sequencing.

### Protein expression, purification and gel filtration

The strain FYJ5210 bearing pET28a::*bioH* was grown to mid-log phase (OD600: ~0.8), and then subjected to 10h of induction with 0.5 mM IPTG at 16°C. The harvested bacterial cells were suspended with lysis buffer [20 mM Tris-HCl (pH8.0), 300 mM NaCl, 5% glycerol, 20 mM imidazole and 1 mM PMSF], and passed through 3 rounds of French Pressure Cell [62]. Following the removal of bacterial debris by the centrifugation at 16800 rpm for 1 h at 4°C, the resultant lysates were incubated with Ni-NTA agarose beads pre- equilibrated with lysis buffer for 1 h [17, 54]. After removing the contaminants, the SUMO-tagged BioH protein was eluted with the elution buffer [20 mM Tris-HCl (pH8.0), 300 mM NaCl, 5% glycerol and 300 mM imidazole]. To liberate the SUMO tag, the fusion protein of BioH was digested with ULPI enzyme in the reaction buffer [20 mM Tris-HCl (pH 8.0), 150 mM NaCl] overnight. The purity of the required BioH protein was determined with 15% SDS-PAGE, and its solution state was determined using gel filtration with a Superdex 75 column.

### *In vitro* enzymatic assays

Because of the commercial unavailability of M-C7-ACP, a cognate substrate for BioH enzyme, the acyl-ACP synthetase (AasS) of *Vibrio harveyi* B132 was utilized to enzymatically synthesize it *in vitro* as earlier described [47, 74]. In this reaction system [100 mM Tris-HCl (pH 7.5), 10 mM MgSO_4_, 10 mM ATP, 5 mM DDT], two reactants (4 μg holo-ACP cargo and 0.4 mM unnatural fatty acid, M-C7) were added in the presence of 0.2 μg AasS enzyme, and maintained at 37°C for 1 h [47]. The BioH reaction system (~20 ul) that consists of 50 mM HEPES buffer (pH 7.0), 5% glycerol, 150 μM M-C7-ACP, and 50 nM BioH, was incubated at 37°C for 0.5 h as we described with little changes [17, 54]. The product C7-ACP was distinguishable from its reactant M-C7-ACP by the separation with conformationally-sensitive gel of 0.5 M urea/PAGE (17.5%, pH9.5) [48–50]. To further identify the C7-ACP product and its substrate M-C7-ACP, the BioH reaction mixtures were dialyzed with 20 mM ammonium acetate overnight at 4°C, and then determined with MALDI-TOF mass spectrometry (Bruker, ultraflextreme) to measure their molecular mass.

### Western blot analysis

To probe the presence of multiple biotinylated proteins, the cell lysates of *P*. *aeruginosa* PAO1 were subjected to the analysis of Western blot using Streptavidin AP-conjugate (Roche) as the primary antibody (1: 1000). The resultant chemiluminescent signals were detected with CDP-Star substrate [75]. Because that the recombinant biotin-requiring enzymes is tagged with N-terminal hexa-histidine, Western blot was carried out using mouse anti-6x his primary antibody, along with the goat anti-mouse IgG secondary antibody, prior to the assay of Streptavidin blot [59, 61].

### Macrophage infections

As Zhu *et al*. described [63], Mouse Bone Marrow-Derived Macrophages (mBMDM) were prepared. Briefly, the cells flushed out from femurs and tibiae of 8-week-old female C57BL/6 mice, were cultured in DMEM media containing 10% heat inactivated fetal bovine serum (FBS), 1% NEAA (Sigma), 1% Na-Pyruvate and 30% supernatant from L929 cells (as source of M-CSF). The cells were Following 5-day incubation at 37°C with 5% CO_2_, the mBMDM cells were harvested. After removal of non-adherent cells by washing with 1x PBS buffer, the adherent cells were plated at 1.5×10^6^ cells/ well, and cultivated overnight prior to the use in infection assays. In general, the mBMDMs were challenged with different bacterial strains (PAO1, Δ*bioH*, and CΔ*bioH*) at a multiplicity of infection (MOI: bacterial counts vs cell counts) of 5. Extracellular bacteria were removed by washing with 1x PBS buffer, following 6h of incubation at 37°C oven containing 5% CO_2_. Then the interested cells were separately harvested at 1h and 24 h post-infection, and lysed with 0.25% SDS. The lysates in appropriate dilution with 1x PBS were plated to measure colony forming unit (CFU). The bacterial survival percentage refers to the ratio of CFU at 24 h in relative to CFU at 1 h.

### Measurement of biotin levels

To measure biotin levels of different origins, the commercial Enzyme-Linked Immunosorbent Assay (ELISA) kit (E-IR-R501, Elabscience) was applied as recommended by the manufacture. The sensitivity and range of this kit was 0.19 ng/ml, and 0.31–20 ng/ml, respectively. It was noted that the wells of Micro ELISA plate were pre-coated with biotin antigen. In general, 50 μl test sample was supplemented into each well of ELISA plate, and then incubated with 50 μl Avidin-HRP working solution for 30 min at 37°C. After 3 rounds of wash, the substrate reagent (90 μl each well) was added, and then incubated for 15 min at 37°C in dark. Finally, the optical density values of samples at the 450 nm wavelength (OD450), were recorded immediately after the addition of 50 μl stop solution. The biotin levels in different samples were calculated by comparing the OD450 values of the samples to a standard four parameter logistic curve.

The samples tested here included i) bacterial lysates of *E*. *coli* MG1655 and *P*. *aeruginosa* PAO1; ii) human and mouse plasma; iii) eight kinds of mouse tissues/organs (heart, liver, spleen, lungs, kidneys, large intestine, small intestine, and stomach); and iv) three types of organ contents (large intestine contents, small intestine contents, and stomach contents). First, the acquired bacterial pellets from *E*. *coli* MG1655 (and/or *P*. *aeruginosa* PAO1) in log-phase were washed three time with 1x PBS buffer, and subjected to the lysis by sonication. After the removal of cell debris by centrifugation, bacterial lysates were produced. Second, the plasma samples of four healthy individuals were kindly provided by Clinical Laboratory of the 2^nd^ Affiliated Hospital (School Hospital Branch), Zhejiang University School of Medicine. The blood collected in anticoagulation tube by mouse tail cutting, proceeded with the 15min of centrifugation (1000×g) at 4°C, and the resultant supernatant was mouse plasma. Third, following the blood collection, mouse was euthanized and then dissected to harvest eight different tissues/organs, exemplified with lungs. To minimize side effects of the hemolyzed blood, mouse tissues were weighed, cut into small pieces, and rinsed in PBS. The acquired tissues were resuspended in appropriate volume of 1x PBS, and fully ground by glass bead. The resultant homogenates were centrifuged for 10 min at 5000×g at 4°C, to generate the supernatant for subsequent biotin quantification. In addition, the contents of three organs (namely large intestine, small intestine, and stomach) were weighed, resuspended in 1ml PBS, and then centrifuged, giving the supernatant used in biotin detection.

When necessary, CD-1 mice (5–6 weeks) were intraperitoneally pre-administered with 2 mg/kg streptavidin to mimic the human environment. Then mouse plasma, tissues, and organ contents were collected at different time courses (1, 2, 4, and 12 h) to determine biotin levels.

### Mouse systemic infections

The model of systemic infection was established by using CD-1 mice (5–6 weeks) intraperitoneally challenged with *P*. *aeruginosa* strains. The bacterial plating on LB agar in series of dilution informed us that OD600 equals to 1x10^9^ CFU for *P*. *aeruginosa*. To remove excess biotin, log-phase culture of *P*. *aeruginosa* was washed three times, and resuspended in 1x PBS of appropriate volume to give 1x10^7^ CFU/ml of inoculums. In general, 15 mice were divided into 3 groups (5 mice each). Apart from the WT, PAO1 strain, Δ*bioH* and its complemented strain CΔ*bioH* were included. As for a standard model, CD-1 mice were intraperitoneally challenged with different strains of *P*. *aeruginosa* (1x10^7^ CFU), and euthanized at 12 h post-infections. If required, CD-1 mice were intraperitoneally administered with 2 mg/kg streptavidin at 1 h before *P*. *aeruginosa* infections. The obtained mouse tissues (heart, liver, spleen, lungs, and kidneys) were weighed, homogenized in 1ml PBS, diluted, and plated on LB agar to calculate the bacterial loads. Eventually, the calculation of Log10(CFU/g), an indicator of bacterial loads, was employed to determine altered infectivity of Δ*bioH* mutant relative to its parental strain PAO1.

### Mouse lung infections

The role of *bioH* in *P*. *aeruginosa* pathogenesis was addressed using the lung infection model of CD-1 mice (5–6 weeks) as recently established by Carfrae *et al*. with minor alteration [15]. In total, four groups of mice (six mice per group) were divided. Apart from the negative control, PBS group, the remaining three groups were separately challenged with the wild-type PAO1, Δ*bioH* mutant, and its complemented strain CΔ*bioH*. Given that excess biotin in mouse plasma occurs at the level, 6-fold higher than that of human plasma, streptavidin (at 2 mg/kg) was administered intraperitoneally prior to mice infection. After the introduction of 5% chloral hydrate as anesthetic by intraperitoneal injection, mice were intratracheally infected at 1x10^7^ CFU of PAO1 and/or its derivatives (Δ*bioH*, and CΔ*bioH*). The survival was recorded during the whole period of 48 h infections, and mice were euthanized at the end of experiment. To better visualize the mouse lung lesions, HE dyeing (i.e., hematoxylin-eosin staining) was conducted. At 12 h post-infections, mouse lung tissues were collected for the preparation of homogenates, and bacterial loads in the lung was calculated by plating. In addition, the ratio of lungs (wet /dry), indicative of pulmonary edema was calculated, because it represents the severity of lung injuries caused by *P*. *aeruginosa*.

### Bioinformatic analyses

Multiple sequence alignment of seven BioH homologs was carried out using ClustalOmega (https://www.ebi.ac.uk/Tools/msa/clustalo), and the output was given via the ESPript 3.0 program (https://espript.ibcp.fr/ESPript/cgi-bin/ESPript.cgi) [76]. In addition to the paradigm *E*. *coli* BioH (b3412) and *P*. *aeruginosa* BioH (PA0502), the remaining 5 orthologs consisted of three BioH-like enzymes (i.e., *V*. *cholerae* vc2718, *Xanthomonas* Xcc0385, plus Lpc_0888 of *Legionella pneumophila*), and two BioHC fusion proteins, namely i) CJA_0428 (502 aa) of *Cellvibrio japonicus*, and ii) Sde_3137 (558 aa) of *Saccharophagus degradans*). The structure of *E*. *coli* BioH (PDB: 1M33) [77] is generated with PyMol (https://pymol.org/2), and the structural model of PA0502 was predicted with Alpha-fold (https://alphafold.ebi.ac.uk) [78, 79].

## Supporting information

S1 Table A collection of biotin-requiring enzymes. (DOCX)

S2 Table Bacterial strains and plasmids used in this study. (DOCX)

S3 Table Primers used in this study. (DOCX)

S1 Fig Sequence alignment of BioH (PA0502) with its related homologs. ClustalOmega (http://www.clustal.org/omega/) was used to conduct sequence alignment. Identical residues are denoted with white letters in red background, similar residues are indicated with dark letters in yellow background, different residues are showed with dark letters, and dots refer to gaps. The catalytic triad of PA0502 consists of S66, D189, and H216 (showed with red arrows). Unlike the paradigm BioH, the *E*. *coli b3412* product, the two homologs (CJA_0428 and Sde_3137) seem to possess an additional domain of BioC. Apart from *E*. *coli* b3412 and *P*. *aeruginosa* PA0502, the remaining five *bioH*-like cousins separately arise from i) the soil bacterium *Cellvibrio japonicus* Ueda107 for CJA_0428, ii) the marine bacterium *Saccharophagus degradans* strain 2–40 for Sde_3137, iii) the plant pathogen *Xanthomonas campestris* for Xcc_0385, iv) the intracellular pathogen *Legionella pneumophila* str. Corby for LPC_0888, and v) the opportunistic pathogen *Vibrio cholerae* N16961 for vc2718.
(TIF)

S2 Fig The monomeric BioH (PA0502) is active in demethylating pimeloyl-ACP methyl ester. **A.** SDS-PAGE (15%) analysis of the purified BioH (PA0502) enzyme. **B.** Size exclusion chromatography analysis of the recombinant BioH (PA0502) protein. The *Streptococcus suis* FakB2 with known size (~33 kDa) [76], is used as a size control here. Using a Superdex 75 column, gel filtration assay unveils the monomeric form of PA0502 (~25 kDa). **C.** The recombinant BioH (PA0502) exhibits the activity of M-C7-ACP demethylation in a dose-dependent manner. **D.** The BioH (PA0502) enzyme (50 nM) displays full activity with M-C7-ACP within 1 min. The conformationally-sensitive gel of 0.5 M urea/17.5% PAGE (pH9.5) was utilized to separate the product C7-ACP from its reactant M-C7-ACP. The symbol of minus “—” denotes no addition of PA0502. The triangle on right hand (panel **C**) denotes varied level of PA0502 protein (ranging from 0, 1, 5, 10, 15, 20, 25, 30 to 35 nM). In contrast, it refers to altered incubation time, varying from 1, 5, 10, 20, 30, 40, 50, 60 to 70 min (panel **D**). Designations: ck, control of holo-ACP; C7-ACP, pimeloyl-ACP; M-C7-ACP, pimeloyl-ACP methyl ester. **E.** A representative MS spectrum of the M-C7-ACP substrate. **F.** The unique MS profile for the product of C7-ACP.
(TIF)

S3 Fig Identification and characterization of BioH (PA0502). **A.** Gel filtration of BioH (PA0502) and its three single mutants. The inside gel verifies the purity of BioH (PA0502) and its derivatives. Size exclusion chromatography analyses indicated that i) the BioH(H216A) mutant remains monomeric, and ii) unlike its wild-type, the two mutants (S66A and D189A) of BioH display the solution structure of polymer. **B.** MS identity of recombinant BioH (PA0502) protein. The polypeptides matched are colored red, and the coverage is 77.50%.
(TIF)

S4 Fig Intraperitoneal administration with streptavidin impacts biotin abundance in different tissues of the infected CD-1 mice. **A.** The time course (0–12 h) of biotin level measured for heart of infected with CD-1 mice post-intraperitoneal administration with streptavidin. **B.** The measured biotin pool of the mouse liver. **C.** The calculated value for biotin pool of the mouse spleen. **D.** The determined level for biotin from the mouse lungs. The measurement of biotin concentration in four different tissues of the infected CD-1 mouse, namely kidneys (**E**), large intestine (**F**), small intestine (**G**), and stomach (**H**). Here, the mouse tissues were sampled at five different time points (0, 1, 2, 4, and 12 h) post-intraperitoneal administration with 2 mg/kg streptavidin as recently Carfrae *et al*. [15] described. All the data are given in means ± SD, and assayed by two-tailed analysis of variance. These findings consistently point to biotin abundance in the above eight kinds of mouse tissues we examined is greatly reduced at 1 h post-intraperitoneal administration with streptavidin.
(TIF)

S5 Fig No effects exerted by BioH on bacterial loads in the routine model of CD-1 mice without the pre-administration with streptavidin. **A.** A scheme of CD-1 mice intraperitoneally challenged with *P*. *aeruginosa* derivatives. Using certain templates freely provided by ScienceSlides Online, we generated the cartoon models. The CD-1 (5–6 weeks) mice are inoculated with 10^7^ CFU of *P*. *aeruginosa*, and euthanized at 12 h post-infection to collect various samples. It was note that, prior to bacterial challenge, CD-1 mice here are not subjected to the intraperitoneal administration with streptavidin. **B.** Minor variation in bacterial loads of the three strains (WT, Δ*bioH*, and CΔ*bioH*) collected from the infected mouse heart. Using the infection model of CD-1 mice without pre-intraperitoneal administration with streptavidin, the impact of BioH on bacterial loads is indistinguishable between WT and its derivatives (Δ*bioH* and CΔ*bioH*). Functional impairment of BioH does not influence bacterial persistence in various mouse tissues, namely liver (**C**), spleen (**D**), lungs (**E**), and kidneys (**F**). The fact that BioH lacks detectable role in bacterial survival within the infected CD-1 mouse reinforced the importance of mimicking the human environment in seeking for the relevance of host biotin to the infectivity of opportunistic pathogen *P*. *aeruginosa* [15]. The data here are presented in averages ± SD, and verified with two-tailed analysis of variance. Designations: WT, the wild-type strain of *P*. *aeruginosa* PAO1; Δ*bioH*, the mutant of *P*. *aeruginosa* devoid of *bioH*; CΔ*bioH*, the genetically-complementary strain of Δ*bioH* mutant.
(TIF)
