# Supplementary material for: The opportunistic pathogen Pseudomonas aeruginosa exploits bacterial biotin synthesis pathway to benefit its infectivity
Source: PLoS Pathog. 2023 Jan 23;19(1):e1011110. doi: 10.1371/journal.ppat.1011110 (PMC9894557; doi:10.1371/journal.ppat.1011110)
Supplement: S1 Table — (DOCX) [file ppat.1011110.s001.docx]

**S1** **Table** A collection of biotin-requiring enzymes

| Locus | Designation | Length (aa) | Motif |
| --- | --- | --- | --- |
| PA1400 | The probable pyruvate carboxylase | 1095 | EAM**K**ME |
| PA2012 | LiuD, α-subunit of methyl-crotonyl-CoA carboxylase | 655 | EAM**K**ME |
| PA2891 | AtuF, α-subunit of geranyl-CoA carboxylase | 661 | EAM**K**ME |
| PA4847 | AccB, biotin carboxyl carrier protein (BCCP) | 156 | EAM**K**MM |
| PA5435 | The probable transcarboxylase subunit | 607 | EAM**K**ME |
