# Supplementary material for: The opportunistic pathogen Pseudomonas aeruginosa exploits bacterial biotin synthesis pathway to benefit its infectivity
Source: PLoS Pathog. 2023 Jan 23;19(1):e1011110. doi: 10.1371/journal.ppat.1011110 (PMC9894557; doi:10.1371/journal.ppat.1011110)
Supplement: S2 Table — (DOCX) [file ppat.1011110.s002.docx]

**S2 Table** Bacterial strains and plasmids used in this study

| Strains/plasmids | Description | Origins | |
| --- | --- | --- | --- |
| Strains | | | |
| DH5α | A cloning host of *E. coli* | Lab stock | |
| BL21 | An expression host of *E. coli* | Lab stock | |
| STL24 | MG1655, Δ*bioH* | Lab stock, (1) |  |
| FYJ517 | MG1655 | Lab stock | |
| Pa-1 | An isolate of pyocyanin-deficient *Pseudomonas* *aeruginosa* (*P. aeruginosa*) with carbapenem resistance | Lab stock | |
| Pa-2 | A clinical isolate of *P. aeruginosa* producing both carbapenem resistance and pyocyanin | Lab stock | |
| FYJ5200 | The PAO1 strain of *Pseudomonas* *aeruginosa* (*P. aeruginosa*) | Lab stock, (2) | |
| FYJ5201 | A *bioH*-deletion mutant of *P*. *aeruginosa* PAO1, Δ*bioH* | This work | |
| FYJ5202 | FYJ5201(PAO1, Δ*bioH*) carrying pAK1900*::bioH* | This work | |
| FYJ5203 | FYJ5201(PAO1, Δ*bioH*) carrying pAK1900*::bioH*(S66A) | This work | |
| FYJ5204 | FYJ5201(PAO1, Δ*bioH*) carrying pAK1900*::bioH*(D189A) | This work | |
| FYJ5205 | FYJ5201(PAO1, Δ*bioH*) carrying pAK1900*::bioH*(H216A) | This work | |
| FYJ5206 | STL24(MG1655, Δ*bioH*) carrying pBAD24*::bioH* | This work | |
| FYJ5207 | STL24(MG1655, Δ*bioH*) carrying pBAD24*::bioH*(S66A) | This work | |
| FYJ5208 | STL24(MG1655, Δ*bioH*) carrying pBAD24*::bioH*(D189A) | This work | |
| FYJ5209 | STL24(MG1655, Δ*bioH*) carrying pBAD24*::bioH*(H216A) | This work | |
| FYJ5210 | BL21 carrying pET28a*::bioH* | This work | |
| FYJ5211 | BL21 carrying pET28a*::bioH*(S66A) | This work | |
| FYJ5212 | BL21 carrying pET28a*::bioH*(D189A) | This work | |
| FYJ5213 | BL21 carrying pET28a*::bioH*(H216A) | This work | |
| FYJ5214 | BL21 carrying pET21a*::*PA1400 | This work | |
| FYJ5215 | BL21 carrying pET21a*::*PA2012 | This work | |
| FYJ5216 | BL21 carrying pET21a*::*PA2891 | This work | |
| FYJ5226 | BL21 carrying pET28a*::*PA4847 | This work | |
| FYJ5228 | BL21 carrying pET28a*::*PA5435 | This work | |
| Plasmids |  |  | |
| pET21a | The T7 promoter-driven expression vector, Amp^R^ | Lab stock | |
| pET28a-Sumo | The T7 promoter-driven expression vector, carrying a sumo tag, Km^R^ | Lab stock | |
| pBAD24 | The arabinose-inducible expression vector, Amp^R^ | Lab stock | |
| pEX18Ap | The SacB-based knockout vector for *P*. *aeruginosa* | Lab stock, (3, 4) | |
| pEX18Ap*::*PA0502-UD | An intermediate plasmid for PA0502 knockout | This work | |
| pEX18Ap*::*PA0502-UGD | The PA0502-knockout plasmid with gentamycin resistance, Gm^R^ | This work | |
| pAK1900 | An *E*. *coli*-*P*. *aeruginosa* shuttle expression vector, Cb^R^ | Lab stock, (3, 5) | |
| pET28a*::bioH* | pET28a encoding *bioH*, Km^R^ | This work | |
| pET28a*::bioH*(S66A) | pET28a encoding the *bioH*(S66A) mutant, Km^R^ | This work | |
| pET28a*::bioH*(D189A) | pET28a encoding the *bioH*(D189A) mutant, Km^R^ | This work | |
| pET28a*::bioH*(H216A) | pET28a encoding the *bioH*(H216A) mutant, Km^R^ | This work | |
| pBAD24*::bioH* | pBAD24 encoding *bioH*, Amp^R^ | This work | |
| pBAD24*::bioH*(S66A) | pBAD24 encoding the *bioH*(S66A) mutant, Amp^R^ | This work | |
| pBAD24*::bioH*(D189A) | pBAD24 encoding the *bioH*(D189A) mutant, Amp^R^ | This work | |
| pBAD24*::bioH*(H216A) | pBAD24 encoding the *bioH*(H216A) mutant, Amp^R^ | This work | |
| pAK1900*::bioH* | pAK1900 encoding *bioH*, Cb^R^ | This work | |
| pAK1900*::bioH*(S66A) | pAK1900 expressing the *bioH*(S66A) mutant, Cb^R^ | This work | |
| pAK1900*::bioH*(D189A) | pAK1900 bearing the *bioH*(D189A) mutant, Cb^R^ | This work | |
| pAK1900*::bioH*(H216A) | pAK1900 encoding the *bioH*(H216A) mutant, Cb^R^ | This work | |
| pET21a*::*PA1400 | pET21a producing PA1400, Amp^R^ | This work | |
| pET21a*::*PA2012 | pET21a expressing PA2012, Amp^R^ | This work | |
| pET21a*::*PA2891 | pET21a bearing PA2891, Amp^R^ | This work | |
| pET28a*::*PA4847 | pET28a encoding PA4847, Km^R^ | This work | |
| pET28a*::*PA5435 | pET28a encoding PA5435, Km^R^ | This work | |

Abbreviations: Amp^R^, Ampicillin resistance; Km^R^, Kanamycin resistance; Gm ^R^, Gentamycin resistance; Cb^R^, Carbenicillin resistance.
