# Supplementary material for: The opportunistic pathogen Pseudomonas aeruginosa exploits bacterial biotin synthesis pathway to benefit its infectivity
Source: PLoS Pathog. 2023 Jan 23;19(1):e1011110. doi: 10.1371/journal.ppat.1011110 (PMC9894557; doi:10.1371/journal.ppat.1011110)
Supplement: S3 Table — (DOCX) [file ppat.1011110.s003.docx]

**S3 Table** Primers used in this study

| Primers | Sequences |
| --- | --- |
| PA0502-up-F(EcoRI) | 5’-TTT GAATTC TCT CCA CCG GCT ACA TGG C-3’ |
| PA0502-up-R(BamHI) | 5’-ACCTCGACGT GGATCC ATC AAC ACG TTC AGG TGC G-3’ |
| PA0502-down-F(BamHI) | 5’-ACGTGTTGAT GGATCC ACG TCG AGG TCT CGA CCC-3’ |
| PA0502-down-R(HindIII) | 5’-TGG AAGCTT GAT GGT GGT CTT GCC GAT C-3’ |
| Gm-GFP-F(BamHI) | 5’-TTT GGATCC CCG GGT ACC GA-3’ |
| Gm-GFP-R(BamHI) | 5’-TTT GGATCC CCG GGT ACC GA-3’ |
| pET28a-*bioH*-F | 5’-GAG AAC AGA TTG GTG GAT CCA TGC GTG ACC ACT TGA TCC T-3’ |
| pET28a-*bioH*-R | 5’-TGG TGG TGG TGG TGC TCG AGT CAG GCA TCG TCA CCC TCG C-3’ |
| *bioH*(S66A)-F | 5’-TGG **GCA** CTG GGA GGC ATG CTC GCC GGC GAA CT-3’ |
| *bioH*(S66A)-R | 5’-ATG CCT CCC AG**T GC**C CAG CCG GCC AGC CAG CT-3’ |
| *bioH*(D189A)-F | 5’-TGT TCG CCG AAG CC**G CA**G CGC TGG TGC CGC TGG CC-3’ |
| *bioH*(D189A)-R | 5’-**TGC** GGC TTC GGC GAA CAG GTG CAA TTG CGG CC-3’ |
| *bioH*(H216A)-F | 5’-AGT **GCA** GGC CTG CCG CTG GAA TGC CCG GAC GA-3’ |
| *bioH*(H216A)-R | 5’-AGC GGC AGG CC**T GC**A CTG GCC GCC AGG GTC GA-3’ |
| pBAD24-*bioH*-F | 5’-TTT TTT GGG CTA GCA GGA GGA ATT CAT GCG TGA CCA CTT GAT CCT-3’ |
| pBAD24-*bioH*-R | 5’-GGT GGT GGT GGT GGT GGT GGT CGA CTC AGG CAT CGT CAC CCT CGC-3’ |
| pAK1900-*bioH*-F | 5’-GTG ACA CTA TAG AAT ACT CAA GCT TAT GCG TGA CCA CTT GAT CCT-3’ |
| pAK1900-*bioH*-R | 5’-GCT CGG TAC CCG GGG ATC CTC TAG ATC AGG CAT CGT CAC CCT CGC-3’ |
| pET21a-PA1400-F | 5’-AAG AAG GAG ATA TAC ATA TGG TGA CCG CTC CCT TCA ACG-3’ |
| pET21a-PA1400-R | 5’-TGG TGG TGG TGG TGC TCG AGT TCG GTC GGT GCC TGC TC-3’ |
| pET21a-PA2012-F | 5’-AAG AAG GAG ATA TAC ATA TGA TGA ACC CGG ACT ACC GCA GCA-3’ |
| pET21a-PA2012-R | 5’-TGG TGG TGG TGG TGC TCG AGG GCC TGG TTC TCG TCC AGC T-3’ |
| pET21a-PA2891-F | 5’-AAG AAG GAG ATA TAC ATA TGA TGC CCA GCT TCA ACA AGA TC-3’ |
| pET21a-PA2891-R | 5’-TGG TGG TGG TGG TGC TCG AGG GCG TCG GCT TCC ACC TC-3’ |
| PA0493-F | 5’-GCG GCA GCC ATA TGG GAT CCA TGG CCG AAC ATA ACG TGC A-3’ |
| PA0493-R | 5’-TGG TGG TGG TGG TGC TCG AGT TAG GTA GCG CTG GCG AT-3’ |
| PA1400-F | 5’-GCG GCA GCC ATA TGG GAT CCA TGG AGT GCC CGC TCG CC-3’ |
| PA1400-R | 5’-TGG TGG TGG TGG TGC TCG AGT TAT TCG GTC GGT GCC TGC-3’ |
| PA2012-F | 5’-GCG GCA GCC ATA TGG GAT CCA TGT TCC TCG AAT GGG AGG G-3’ |
| PA2012-R | 5’-TGG TGG TGG TGG TGC TCG AGT TAG GCC TGG TTC TCG TCC-3’ |
| PA2891-F | 5’-GCG GCA GCC ATA TGG GAT CCA TGC TCT GGC TAT ACG GCC-3’ |
| PA2891-R | 5’-TGG TGG TGG TGG TGC TCG AGT TAG GCG TCG GCT TCC AC-3’ |
| PA4847-F | 5’-GCG GCA GCC ATA TGG GAT CCA TGT ACG CAC AGG CTC CGG-3’ |
| PA4847-R | 5’-TGG TGG TGG TGG TGC TCG AGT TAG ACG ATG GTG AAC AGC G-3’ |
| PA5435-F | 5’-GCG GCA GCC ATA TGG GAT CCA TGG ACG GCA TGC CGG AAG-3’ |
| PA5435-R | 5’-TGG TGG TGG TGG TGC TCG AGT TAG CCC GCA ATC TCG ATC A-3’ |
| KPC-2-F | 5’- CAG CTC ATT CAA GGG CTT TCT T -3’ |
| KPC-2-R | 5’- GCA GAC TCC AGC CTA AAT GTG -3’ |

The underlined letters refer to the restriction cuts. The bold letters denote the codons with a certain mutation.
