# Supplementary figures and images for: The opportunistic pathogen Pseudomonas aeruginosa exploits bacterial biotin synthesis pathway to benefit its infectivity
Source: PLoS Pathog. 2023 Jan 23;19(1):e1011110. doi: 10.1371/journal.ppat.1011110 (PMC9894557; doi:10.1371/journal.ppat.1011110)

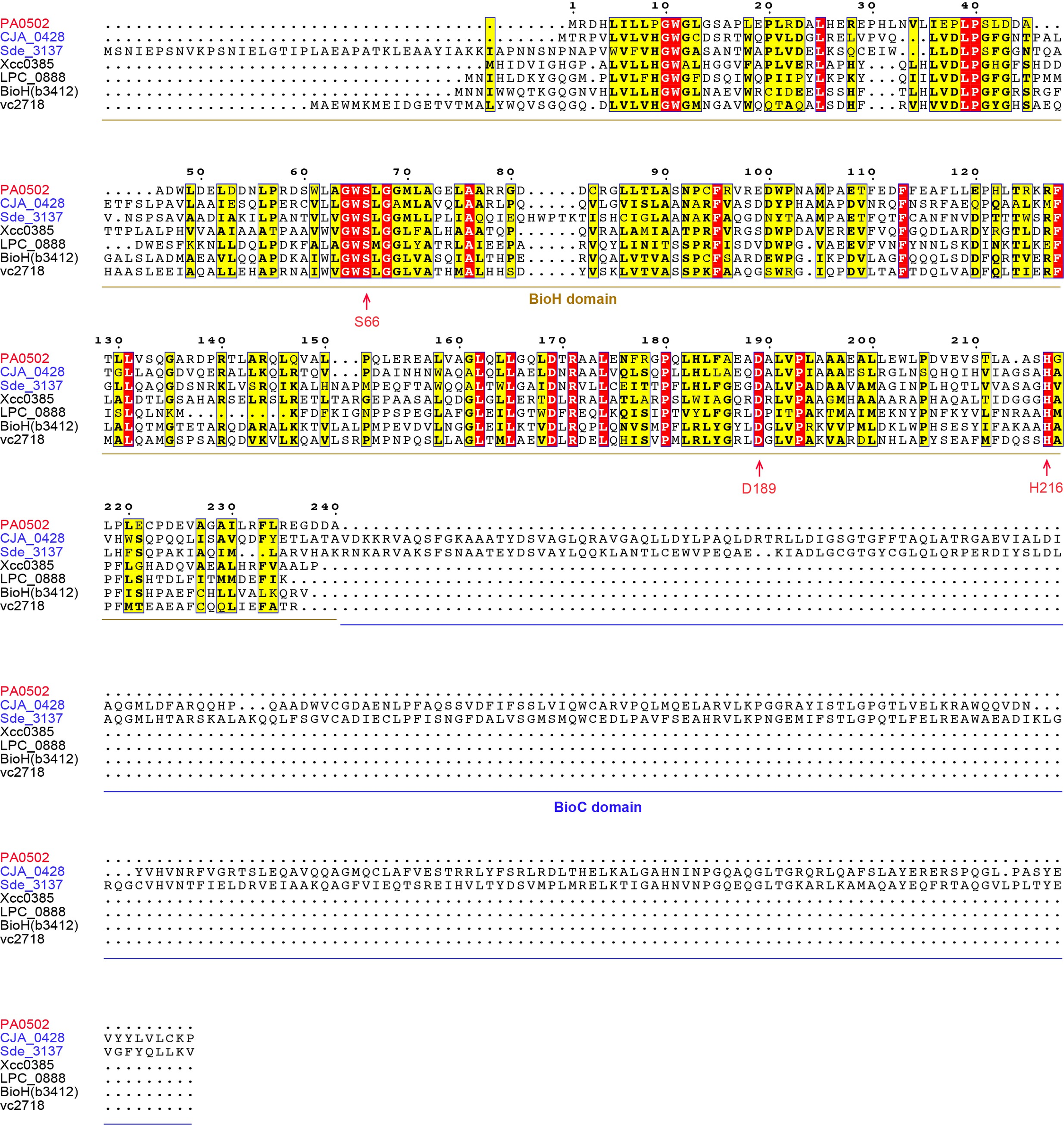

Supplement: S1 Fig — ClustalOmega (http://www.clustal.org/omega/) was used to conduct sequence alignment. Identical residues are denoted with white letters in red background, similar residues are indicated with dark letters in yellow background, different residues are showed with dark letters, and dots refer to gaps. The catalytic triad of PA0502 consists of S66, D189, and H216 (showed with red arrows). Unlike the paradigm BioH, the E. coli b3412 product, the two homologs (CJA_0428 and Sde_3137) seem to possess an additional domain of BioC. Apart from E. coli b3412 and P. aeruginosa PA0502, the remaining five bioH-like cousins separately arise from i) the soil bacterium Cellvibrio japonicus Ueda107 for CJA_0428, ii) the marine bacterium Saccharophagus degradans strain 2–40 for Sde_3137, iii) the plant pathogen Xanthomonas campestris for Xcc_0385, iv) the intracellular pathogen Legionella pneumophila str. Corby for LPC_0888, and v) the opportunistic pathogen Vibrio cholerae N16961 for vc2718. (TIF) [file ppat.1011110.s004.tif]

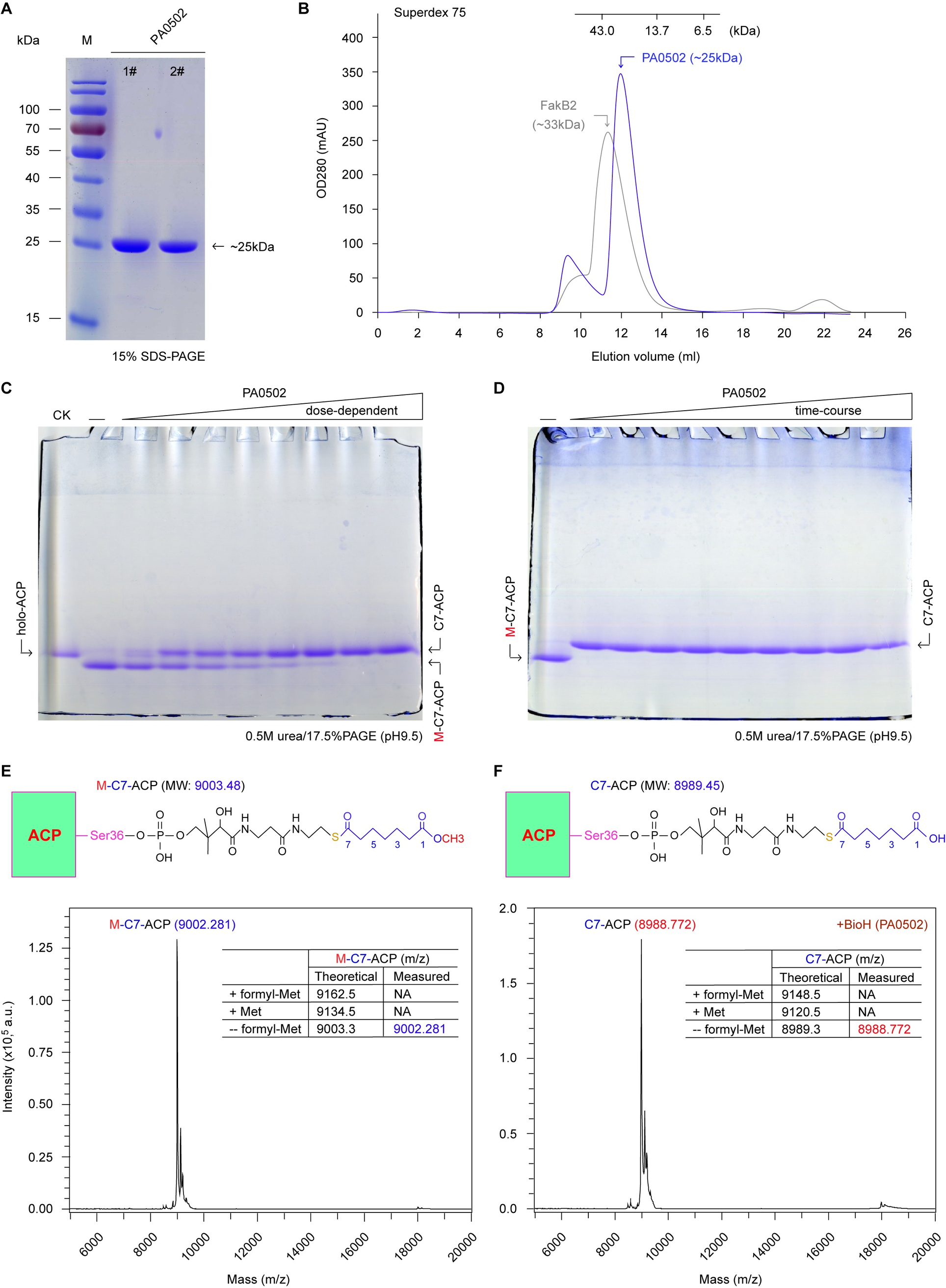

Supplement: S2 Fig — A. SDS-PAGE (15%) analysis of the purified BioH (PA0502) enzyme. B. Size exclusion chromatography analysis of the recombinant BioH (PA0502) protein. The Streptococcus suis FakB2 with known size (~33 kDa) [76], is used as a size control here. Using a Superdex 75 column, gel filtration assay unveils the monomeric form of PA0502 (~25 kDa). C. The recombinant BioH (PA0502) exhibits the activity of M-C7-ACP demethylation in a dose-dependent manner. D. The BioH (PA0502) enzyme (50 nM) displays full activity with M-C7-ACP within 1 min. The conformationally-sensitive gel of 0.5 M urea/17.5% PAGE (pH9.5) was utilized to separate the product C7-ACP from its reactant M-C7-ACP. The symbol of minus “—” denotes no addition of PA0502. The triangle on right hand (panel C) denotes varied level of PA0502 protein (ranging from 0, 1, 5, 10, 15, 20, 25, 30 to 35 nM). In contrast, it refers to altered incubation time, varying from 1, 5, 10, 20, 30, 40, 50, 60 to 70 min (panel D). Designations: ck, control of holo-ACP; C7-ACP, pimeloyl-ACP; M-C7-ACP, pimeloyl-ACP methyl ester. E. A representative MS spectrum of the M-C7-ACP substrate. F. The unique MS profile for the product of C7-ACP. (TIF) [file ppat.1011110.s005.tif]

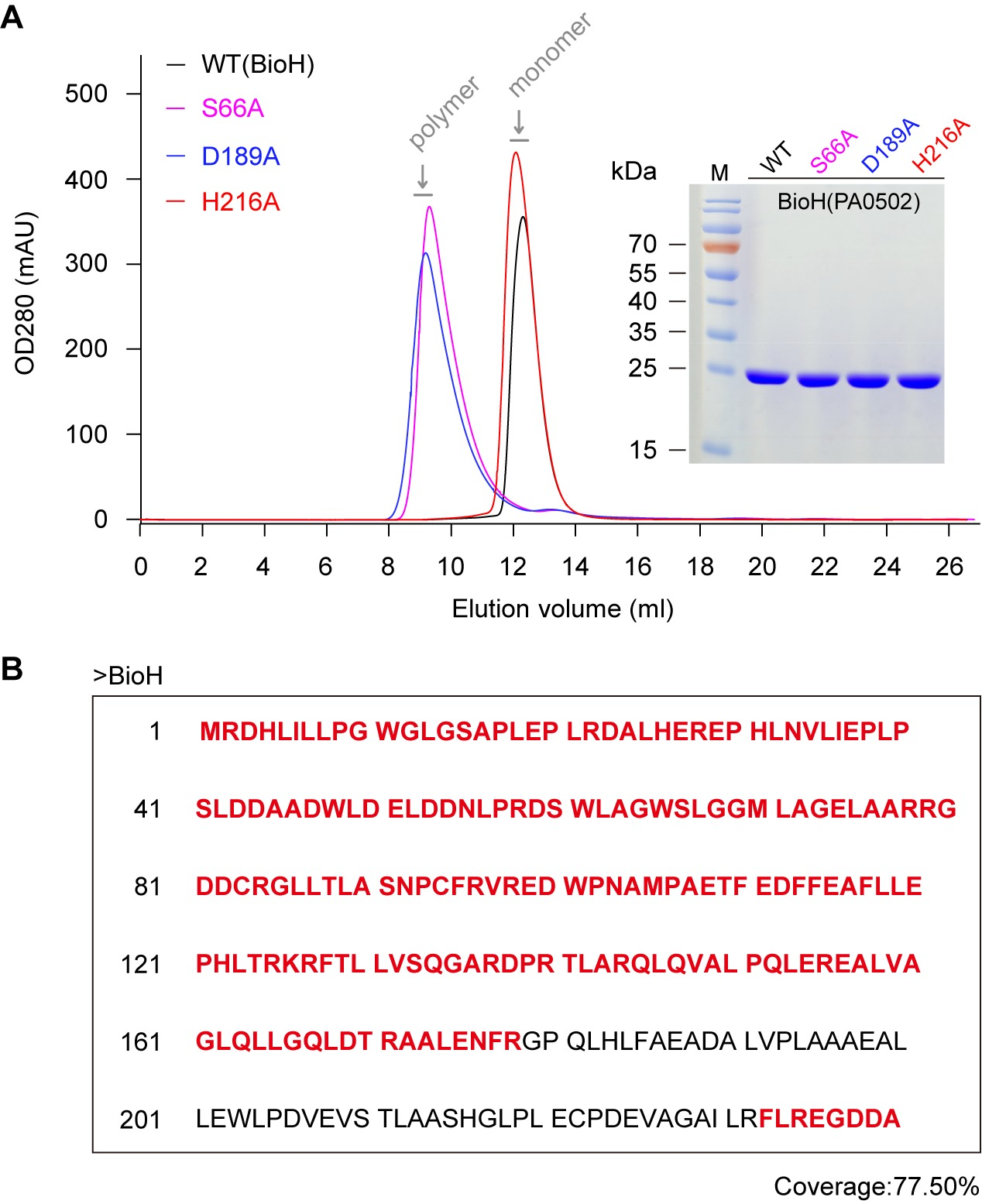

Supplement: S3 Fig — A. Gel filtration of BioH (PA0502) and its three single mutants. The inside gel verifies the purity of BioH (PA0502) and its derivatives. Size exclusion chromatography analyses indicated that i) the BioH(H216A) mutant remains monomeric, and ii) unlike its wild-type, the two mutants (S66A and D189A) of BioH display the solution structure of polymer. B. MS identity of recombinant BioH (PA0502) protein. The polypeptides matched are colored red, and the coverage is 77.50%. (TIF) [file ppat.1011110.s006.tif]

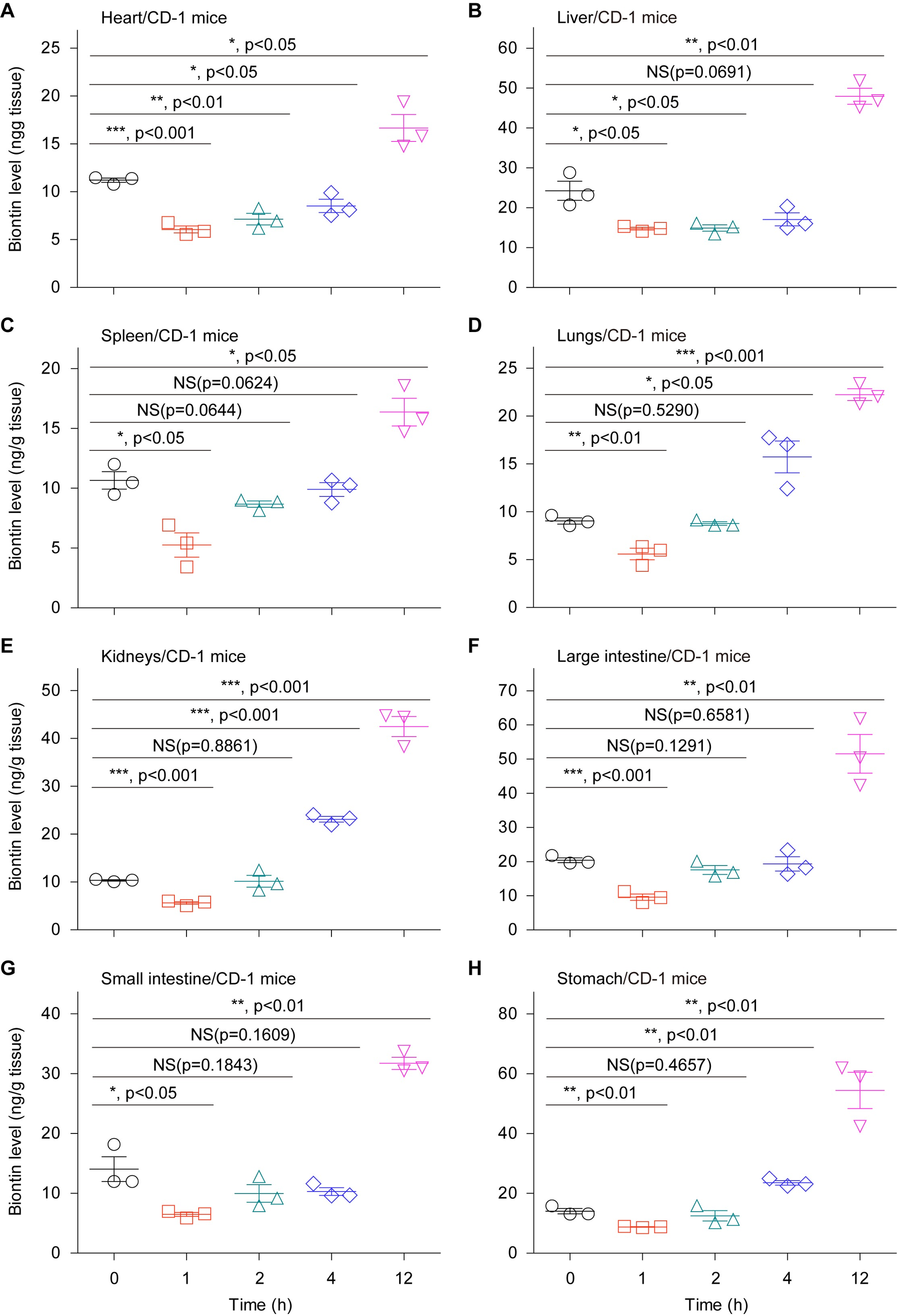

Supplement: S4 Fig — A. The time course (0–12 h) of biotin level measured for heart of infected with CD-1 mice post-intraperitoneal administration with streptavidin. B. The measured biotin pool of the mouse liver. C. The calculated value for biotin pool of the mouse spleen. D. The determined level for biotin from the mouse lungs. The measurement of biotin concentration in four different tissues of the infected CD-1 mouse, namely kidneys (E), large intestine (F), small intestine (G), and stomach (H). Here, the mouse tissues were sampled at five different time points (0, 1, 2, 4, and 12 h) post-intraperitoneal administration with 2 mg/kg streptavidin as recently Carfrae et al. [15] described. All the data are given in means ± SD, and assayed by two-tailed analysis of variance. These findings consistently point to biotin abundance in the above eight kinds of mouse tissues we examined is greatly reduced at 1 h post-intraperitoneal administration with streptavidin. (TIF) [file ppat.1011110.s007.tif]

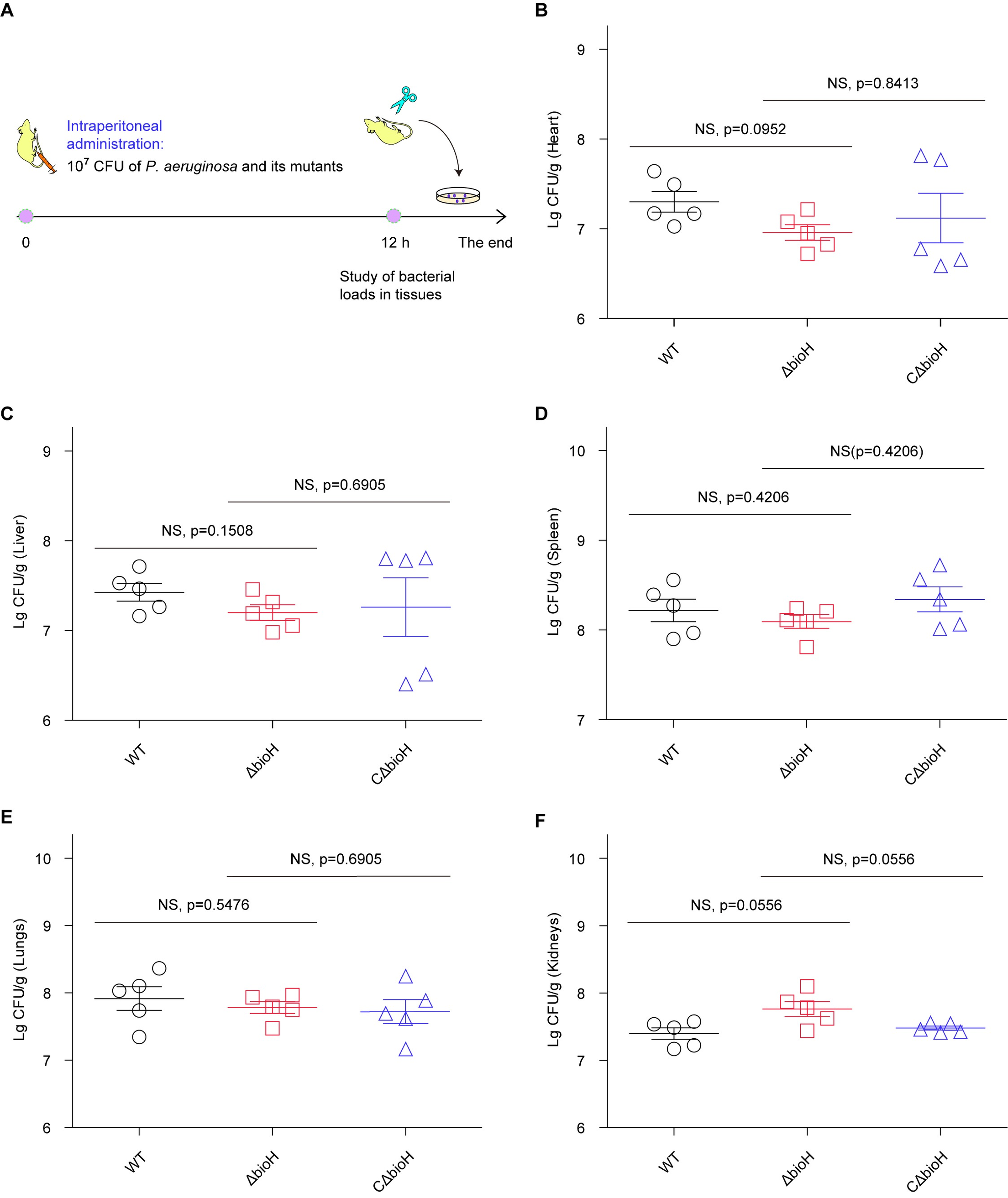

Supplement: S5 Fig — A. A scheme of CD-1 mice intraperitoneally challenged with P. aeruginosa derivatives. Using certain templates freely provided by ScienceSlides Online, we generated the cartoon models. The CD-1 (5–6 weeks) mice are inoculated with 107 CFU of P. aeruginosa, and euthanized at 12 h post-infection to collect various samples. It was note that, prior to bacterial challenge, CD-1 mice here are not subjected to the intraperitoneal administration with streptavidin. B. Minor variation in bacterial loads of the three strains (WT, ΔbioH, and CΔbioH) collected from the infected mouse heart. Using the infection model of CD-1 mice without pre-intraperitoneal administration with streptavidin, the impact of BioH on bacterial loads is indistinguishable between WT and its derivatives (ΔbioH and CΔbioH). Functional impairment of BioH does not influence bacterial persistence in various mouse tissues, namely liver (C), spleen (D), lungs (E), and kidneys (F). The fact that BioH lacks detectable role in bacterial survival within the infected CD-1 mouse reinforced the importance of mimicking the human environment in seeking for the relevance of host biotin to the infectivity of opportunistic pathogen P. aeruginosa [15]. The data here are presented in averages ± SD, and verified with two-tailed analysis of variance. Designations: WT, the wild-type strain of P. aeruginosa PAO1; ΔbioH, the mutant of P. aeruginosa devoid of bioH; CΔbioH, the genetically-complementary strain of ΔbioH mutant. (TIF) [file ppat.1011110.s008.tif]
